# A Tale That Morphology Fails to Tell: A Molecular Phylogeny of Aeolidiidae (Aeolidida, Nudibranchia, Gastropoda)

**DOI:** 10.1371/journal.pone.0063000

**Published:** 2013-05-02

**Authors:** Leila Carmona, Marta Pola, Terrence M. Gosliner, Juan Lucas Cervera

**Affiliations:** 1 Departamento de Biología, Facultad de Ciencias del Mar y Ambientales, Campus de Excelencia Internacional del Mar (CEI·MAR), Universidad de Cádiz, Puerto Real, Cádiz, Spain; 2 Departamento de Biología, Edificio de Biología, Campus de Excelencia Internacional UAM+CSIC, Universidad Autónoma de Madrid, Madrid, Spain; 3 Department of Invertebrate Zoology, California Academy of Sciences, San Francisco, California, United States of America; J. Craig Venter Institute, United States of America

## Abstract

Aeolidida is one of the largest clades of nudibranchs with at least 560 known species. However, its systematics has not been studied in a comprehensive manner. Phylogenetic analyses of larger clades such as Nudibranchia or Cladobranchia have usually included a poor sample of aeolids. Furthermore, phylogenetic studies at the family or generic level in Aeolidida are a few and far between. The first molecular phylogeny of the aeolid family Aeolidiidae is presented here. This study, the most comprehensive for Aeolidida to date, uses new sequences of two mitochondrial (COI and 16S) genes and one nuclear gene (H3). 251 specimens from members of seven families of Aeolidida, including 39 species of Aeolidiidae were studied. Excluding *Pleurolidia juliae*, Aeolidiidae is monophyletic. Our results resolve the systematic relationships within the Aeolidiidae at a generic level, requiring changes in the systematics of this family. *Spurilla, Anteaeolidiella, Limenandra* and *Aeolidia* are well-supported and monophyletic clades. *Aeolidiella stephanieae* is transferred to *Berghia* and *Aeolidiopsis ransoni* and *Spurilla salaamica* to *Baeolidia*, to maintain the monophyletic lineages reflected in this study. The systematics of *Cerberilla* remains unclear. Some species earlier attributed to *Aeolidiella* are now grouped in a previously unnamed clade that we designate as *Bulbaeolidia* gen. nov.

## Introduction

Aeolidida contains about a dozen families constituted by approximately 560 described species [1]. Members of this taxon are easily distinguished by their elongated and tapering bodies, lacking distinct gills. All members have specialized dorsal appendages, called cerata, that are used in respiration and defence. The cerata contain branches of the digestive gland that transports nematocysts (acquired from the prey) to their tips where they are stored in the so-called cnidosacs. The vast majority of the aeolids possess a uniseriate radula, although some genera also have lateral teeth (e.g. *Flabellina* spp, *Eubranchus* spp, *Notaeolidia* spp). Although it constitutes the second largest group of Nudibranchia [2], few phylogenetic studies have been undertaken so far. Several contributions are focused on some aeolid genera or families [3]–[13]. Some of them also include phylogenetic analyses, but only Moore & Gosliner [12] and Carmona et al. [13] employed a molecular approach. Most of the phylogenetic studies focused on higher taxa, such as Heterobranchia, Euthyneura, Opisthobranchia, Nudibranchia or Cladobranchia include some aeolid species [14]–[26]. These studies support the monophyly of the aeolids, identifying the presence of cnidosacs and the transformation of the oral veil into oral tentacles as synapomorphies [26]. Only Martin et al. [27] rejected the monophyletic status of Aeolidida after including *Hancockia* and *Embletonia*.

Within Aeolidida, Aeolidiidae is one of the largest families, whose members differ from the rest of aeolids by their pectinate radular teeth [4], [8], [28]–[30] and their diet –most of them feed on anemones. Indeed, many species sequester zooxanthellae from their prey and maintain the dinoflagellates alive in their tissues in order to use the photosynthetic products for their own nutrition (e.g. *Spurilla neapolitana*, *Berghia verrucicornis*, *B. coerulescens*, *“S. salaamica”*,*“B. major”*, *Aeolidiella alderi*, *“A. stephanieae”*; see Kempf [31] and Marín & Ros [32]). This family is composed of the following genera: *Aeolidia* Cuvier, 1798; *Spurilla* Bergh, 1864; *Aeolidiella* Bergh, 1867; *Cerberilla* Bergh, 1873; *Berghia* Trinchese, 1877; *Baeolidia* Bergh, 1888; *Protaeolidiella* Baba, 1955; *Aeolidiopsis* Pruvot-Fol, 1956; *Limenandra* Haefelfinger & Stamm, 1958; *Anteaeolidiella* Miller, 2001; *Burnaia* Miller, 2001 and *Milleraeolidia* Ortea, Caballer & Espinosa, 2004, although the validity of some of these taxa has been questioned [4], [8], [33]. Furthermore, the proposed wide geographical distribution for several species (*Aeolidia papillosa*, *Anteaeolidiella indica*, *Berghia coerulescens*, *Limenandra nodosa*, *Spurilla neapolitana*) is controversial and raises doubts about the conspecificity of the different populations that are geographically isolated.

Recent morphological and molecular approaches provided a preliminary idea about the phylogenetic relationships between Aeolidiidae and other members of Aeolidida [10], [13]. The systematic relationships between genera and/or species of Aeolidiidae itself have been subject of controversy for the last seventy years [3]–[4], [8], [30], [34]–[43]. In 1939, based on differences in the position of the nephroproct, Odhner [27] removed *Spurilla* and *Berghia* from Aeolidiidae and placed them in a new family called Spurillidae. Haefelfinger & Stamm [35] erected the genus *Limenandra* with *L. nodosa* as the type species and transferred *Baeolidia fusiformis* Baba, 1949 to this new genus. Ten years later, Odhner (in [37]) included *Baeolidia* in Spurillidae but overlooked *Limenandra*. Gosliner [41] regarded *Limenandra* as a junior synonym of *Baeolidia* and Rudman [30] considered *Berghia* as a junior synonym of *Spurilla*. In the same year, Schmekel & Portmann [42], considering only the Mediterranean species, split Aeolidiidae in two subfamilies: Aeolidiinae (*Aeolidiella*) and Spurillinae (*Spurilla, Berghia* and *Limenandra*). In 1985, Gosliner [4] used his previous classification with the aeolidiids from South Africa. Five years later, Rudman [44] regarded *Pleurolidia* as a junior synonym of *Protaeolidiella* and considered *Protaeolidiella atra* Baba, 1955 and *Pleurolidia juliae* Burn, 1966 to be conspecific. Finally, in the 21st century *Anteaeolidiella, Burnaia* and *Milleraeolidia* were included within Aeolidiidae [8], [45], although *Milleraeolidia ritmica* Ortea, Caballer & Espinosa 2004, the sole species of this genus, has been considered as a synonym of *Berghia creutzbergi* (Marcus & Marcus, 1970) by Valdés et al. [46] and García-García et al. [33].

The primary objective of this study is to elucidate the phylogenetic relationships of the family Aeolidiidae. Molecular phylogenetic analyses were undertaken based on two mitochondrial and one nuclear gene (mitochondrial COI and 16S rRNA, and nuclear H3). Representatives of seven currently recognized families of Aeolidida have been included. The specific aims are: (i) to test if “Spurillidae”/“Spurillinae” and “Aeolidiinae” are monophyletic; (ii) to test the monophyly of the different genera of Aeolidiidae, reviewing their phylogenetic relationships.

## Materials and Methods

### Sampling

Samples were obtained using standard SCUBA diving sampling techniques for opisthobranchs and through the study of museum collections. Two hundred and fifty-one specimens including 39 nominal species of Aeolidiidae, 3 species of Babakinidae, 33 species of Facelinidae, 7 species of Flabellinidae, 1 species of Fionidae, 3 species of Piseinotecidae and 4 species of Tergipedidae were studied. The classification of all the species used in this study is listed in Table S1 and arranged based on Gosliner et al. [47] classification. Numbers following “sp.” in the names of undescribed species refer to the identification system used by Gosliner et al. [47]. Undescribed species labelled as “sp.” followed by a capital letter refer to undescribed species not included in Gosliner et al. [47]. Voucher specimens are held in the collections of the California Academy of Sciences, CASIZ (San Francisco, USA), Museo Nacional de Ciencias Naturales, MNCN (Madrid, Spain), Museu de Zoologia da Universidade São Paulo, MZSP (São Paulo, Brazil), Museu Nacional/Universidade Federal do Rio de Janeiro, MNRJ (Rio de Janeiro, Brazil), University Museum of Bergen, ZMBN (Bergen, Norway) or Zoologische Staatssammlung München, ZSM (Munich, Germany).

### Ethic Statements

The majority of the specimens used in this study belongs to the California Academy of Sciences Invertebrate Zoology (CASIZ) collection and the DNA collection of the Museo Nacional de Ciencias Naturales (MNCN) of Madrid. We had the permission to take tissue samples from all the specimens for DNA analyses, regardless where the specimen was deposited. As stated in the CASIZ collections policy: ‘No specimens will be accessioned without adequate labelling, collection notes, field notes, or other locality information, nor without appropriate legal documentation (collecting permits, export permits from country of origin, etc.) when applicable.’ Those DNA samples deposited in the MNCN were obtained from pieces of foot from specimens collected during field trips. These specimens will provisionally remain at the University of Cádiz until their anatomical study is completed. Thereafter, each specimen will be deposited in the MNCN. All the museums listed in point 2.1, from which material has been examined in the present study, have strict collection policies in order to avoid illegal practices. Donors are required to provide all the available information about their field studies, including collecting and export permits from country of origin, as well as the number of species and specimens exported. Therefore, the legality of all this material is assumed. On the other hand, all necessary permits were obtained for the field studies carried out in Cuba, Bermuda, Azores, Madeira, Chafarinas Is. and Balearic Is. by the authors. These permits were facilitated by Cuban environmental authorities through the Centro de Investigaciones Marinas (C. I. M.) of the University of La Habana, by the Department of Environmental Protection of the Government of Bermuda, by Secretaria Regional do Ambiente e do Mar of the Regional Government of Azores, by the Secretaria Regional do Ambiente e dos recursos Naturais of the Regional Government of Madeira, by the Organismo Autónomo de Parques Nacionales (Government of Spain) for the Chafarinas Islands and the Dirección General de Pesca of the Regional Government of Balearic Islands. For the remaining localities (Japan, Sweden, France, Morocco, Canary Is., Senegal, Cape Verde, Greece and Italy), the specimens were collected and sent by several gracious collaborators. These locations were not privately-owned or legally protected. Finally, none of the studied species are protected, listed as endangered, or included in the CITES list.

### DNA Extraction, Amplification and Sequencing

A total of 194 specimens were successfully sequenced for the cytochrome *c* oxidase subunit I (COI), 228 for the 16S rRNA (16S) and 229 for the Histone-3 (H3) genes. Thirty-two additional sequences were obtained from GenBank (see Table S1 for full list of samples, localities, and voucher references).

*Tritonia antarctica* was chosen as the outgroup, due to its basal placement within Cladobranchia [23].

Excluding *Anteaeolidiella* (see *Anteaeolidiella* discussion), *Baeolidia* and *Cerberilla*, the identities of the different clades within Aeolidiidae were conducted based on the type species of each genus. For those inconclusive clades, current names were retained. In order to minimize disruption to nomenclature, synonyms were resurrected whenever possible.

DNA was extracted from foot tissue of specimens preserved with 70–100% ethanol, except in the cases of small individuals that were used whole. The DNeasy Blood and Tissue Kit of Qiagen (Qiagen, Valencia, CA, USA; 09/2001) was used for DNA extraction.

Partial sequences of COI, 16S and H3 were amplified by polymerase chain reaction (PCR) using the primers: LCO1490 (5′-GGTCAACAAATCATAAAGATATTGG-3′) and HCO2198 (5′-TAAACTTCAGGGTGACCAAAAATCA-3′) [48] for COI; 16S ar-L (5′-CGCCTGTTTATCAAAAACAT-3′) and 16S br-H (5′-CCGGTCTGAACTCAGATCACGT-3′) [49] for 16S rRNA; and H3AD5′3′ (5′- ATGGCTCGTACCAAGCAGACVGC-3′) and H3BD5′3′ (5′-ATATCCTTR GGCATRATRGTGAC-3′) [50] for H3. These three gene regions are commonly used in systematic studies of gastropods (e.g. [23], [51]–[55]). However, several internal primers for COI and H3 were designed for those specimens that could not be amplified using the universal ones (Table 1).

**Table 1:** Forward (F) and reverse (R) PCR specific primers.

Name	Sequence 5′-3′
COI	
Apapi_COI_IntF	TGTGGTGTGGATTAGCAGGA
Apapi_COI_IntR	CAGCCAAAACCGGTAAAGAT
Bergh_COI_IntF	ATTRGGAATGTGATGTGGGT
Bergh_COI_IntR	CCAGCAGGRTCRAAAAACCT
Lime_COI_IntrF	TGTTTTAYTAGGRATGTGATGTGG
Lime_COI_IntrR	TTGTAGTAATAAAATTAATTGCCCCA
Snea_COI_IntF	TTCGTTTTGAACTYGGAACRG
Snea_COI_IntR	CACCAGCTAAAACAGGTAGMG
H3	
Apapi_H3_IntF	TAAATCCACCGGAGGAAAGG
Apapi_H3_IntR	CCTCAANCAGACCGACCAAG

PCRs were conducted in a 50 µl volume reactions containing 2 µl of both forward and reverse primers (10 µM), 5 µl of dNTP (2 mM), a gene-dependent amount of magnesium chloride (25 mM), 0.5 µl of Qiagen DNA polymerase (250 units), 10 µl of “Q-solution” (5×), and 5 µl of Qiagen buffer (10×) (Qiagen Taq PCR Core Kit cat. no. 201225). Magnesium chloride amounts were 7 µl for COI and 16S, and 4 µl for H3. Amplification of COI was performed with an initial denaturation for 5 min at 94°C, followed by 35 cycles of 1 min at 94°C, 30 s at 44°C (annealing temperature) and 1 min at 72°C with a final extension of 7 min at 72°C. The 16S amplification began with an initial denaturation for 5 min at 95°C followed by 35 cycles of 30 s at 94°C, 30 s at 44°C (annealing temperature), 1 min at 72°C with a final extension of 7 min at 72°C. H3 amplification was performed with an initial denaturation for 3 min at 95°C, followed by 40 cycles of 45 s at 94°C, 45 s at 50°C (annealing temperature), 2 min at 72°C, with a final extension of 10 min at 72°C.

Successful PCR product was purified mixing 5 µl of PCR product with 2 µl of ExoSAP-IT (usb.affymetrix.com). Samples were incubated at 37°C for 15 min followed by an inactivation step at 80°C for 15 min Sequence reactions were run on a 3730XL DNA sequencer, Applied Biosystems. All new sequences have been deposited in GenBank.

### Sequence Alignment and Phylogenetic Analyses

DNA sequences were assembled and edited using Geneious Pro 4.7.6 [56]. All the sequences were checked for contamination with BLAST [57] implemented in the Genbank database. Geneious and MAFFT [58] were employed to align the sequences, using the default settings in both programs. The alignments were checked by eye using MacClade (version 4.06) [59]. Protein-coding sequences were translated into amino acids for alignment confirmation. Saturation was visually inspected in MEGA 5.0 [60] by plotting for all specimens including outgroup the total number of pairwise differences (transitions and transversions) against uncorrected *p*-distances. For the COI and H3 genes, saturation was further examined separately for the first, second and third codon positions.

The most variable regions from the 16S rRNA alignment were removed using both the default settings and the standard options for stringent and less stringent selection in Gblocks [61]. Excluding “indel-rich” regions, the tree was in general poorly resolved with lower node support. Therefore, final analyses were performed including all bases. Sequences of COI, 16S, and H3 were trimmed to 669, 484, and 328 base pairs, respectively.

Individual gene analyses and a concatenated analysis were performed. To test for conflicting phylogenetic signal between genes, the incongruence length difference test (ILD) [62] was conducted as the partition homogeneity test in PAUP* 4.0b10 [63]. Test settings consisted of 10 random stepwise additions (100 replicates) with TBR branch swapping.

The best-fit models of evolution for each gene were determined using the Akaike information criterion [64] implemented in MrModeltest 2.3 [65]. The GTR+I+G was selected for the three genes.

Maximum likelihood (ML) analyses were performed using the software RAxML v7.0.4 [66] and node support was assessed with non-parametric bootstrapping (BS) with 60000 replicates, random starting trees, and parameters estimated from each dataset under the model selected for the original dataset. Bayesian inference analyses (BI) were conducted using MrBayes version 3.1.2b [67] for thirty nine million generations and four chains. Markov chains were sampled every 1000 generations.

The models implemented were those estimated with MrModeltest 2.3. The combined dataset was partitioned among genes and the “unlink” command was used to allow all parameters to vary independently within each partition.

Convergence was diagnosed graphically by plotting for each run the likelihood against the number of generations using the software Tracer version 1.4.1 [68]. For each analysis the first 15000 trees were discarded (‘burn-in’ period) and node support was assessed with posterior probabilities (PP). Only nodes supported by BS ≥75 and PP≥0.90 are discussed. All the alignments and trees published in this study are deposited in TreeBase under project number S14014 (http://purl.org/phylo/treebase/phylows/study/TB2:S14014).

### Genetic Distances

In order to compare the genetic distances amongst specimens of Aeolidiidae, we calculated the pairwise uncorrected *p*-distances for COI using PAUP* 4.0 b 10.0. (Table 2). All codon positions were considered for the analysis.

**Table 2:** Minimum COI gene pairwise uncorrected *p*-distances amongst key species.

Species			COI genetic distances (%)
*A. lurana* Naples	vs.	*A. lurana* Brazil	0
*A. papillosa* Sweden	vs.	*A. papillosa* Maine	0.3
*L. nodosa* France	vs.	*L. nodosa* Bahamas	1
*S. braziliana* Costa Rica (PAC)	vs.	*S. braziliana* Japan	1.9
*B. rissodominguezi* Cuba	vs.	*B. stephanieae* Florida	5.7
*S. neapolitana* Naples	vs.	*S. sargassicola* Bahamas	6.4
*Limenandra* sp. A Mexico (PAC)	vs.	*L. nodosa* Bahamas	7.3
*A. papillosa* WA	vs.	*Aeolidia* sp. B California	8.4
*A. saldanhensis* South Africa	vs.	*A. lurana* Brazil	8.4
*S. braziliana* Costa Rica (PAC)	vs.	*Spurilla* sp. A Morocco (ATL)	10.8
*A. papillosa* Maine	vs.	*Aeolidia* sp. A France (ATL)	11
*A. saldanhensis* South Africa	vs.	*A. cacaotica* Australia	11.7
*A. lurana* Naples	vs.	*A. cacaotica* Australia	11.8
*A. takanosimensis* Japan	vs.	*A. lurana* Brazil	12.4
*B. japonica* Marshall Is.	vs.	*Baeolidia* sp. C Marshall Is.	12.6
*A. saldanhensis* South Africa	vs.	*A. takanosimensis* Japan	13
*A. takanosimensis* Japan	vs.	*A. cacaotica* Australia	13
*Aeolidia* sp. A France (ATL)	vs.	*Aeolidia* sp. B California	13.3
*B. alba* Malaysia	vs.	*Bulbaeolidia* sp. A Brazil	13.5
*S. braziliana* Japan	vs.	*S. sargassicola* Bahamas	13.6
*S. neapolitana* Portugal	vs.	*S. braziliana* Brazil	14.1
*A. takanosimensis* Japan	vs.	*Anteaeolidiella* sp. A Clipperton Is.	14.2
*S. sargassicola* Bahamas	vs.	*Spurilla* sp. A Morocco (ATL)	14.8
*S. neapolitana* Naples	vs.	*Spurilla* sp. A Morocco (ATL)	14.8

Names are final identifications after the analyses.

ATL = Atlantic Ocean;

PAC = Pacific Ocean;

WA = western Atlantic Ocean.

### Species Concept and Genetic Divergence Thresholds

To define species, we used the criteria of divergence and reciprocal monophyly supported by independent genetic markers [55], [69]–[72]. Since different groups of organisms may present distinct rates of evolution, the use of genetic threshold is difficult to apply [73]–[74]. However, based on our molecular as well as our morphology results, we established a cut-off range of 5.5–16% between sister species of Aeolidiidae (uncorrected *p*-distance for COI gene). Table S2 shows the minimum uncorrected *p*-distance for COI between sister species of each genus.

### Nomenclatural Acts

The electronic edition of this article conforms to the requirements of the amended International Code of Zoological Nomenclature, and hence the new names contained herein are available under that Code from the electronic edition of this article. This published work and the nomenclatural acts it contains have been registered in ZooBank, the online registration system for the ICZN. The ZooBank LSIDs (Life Science Identifiers) can be resolved and the associated information viewed through any standard web browser by appending the LSID to the prefix "http://zoobank.org/". The LSID for this publication is: LSID urn:lsid:zoobank.org:pub:33A960E8-2106-41DF-A5C8-4FA68B0974CB. The electronic edition of this work was published in a journal with an ISSN, and has been archived and is available from the following digital repositories: It will be available on the Research website of the California Academy of Sciences http://research.calacademy.org/.

## Results

**Figure 1:**
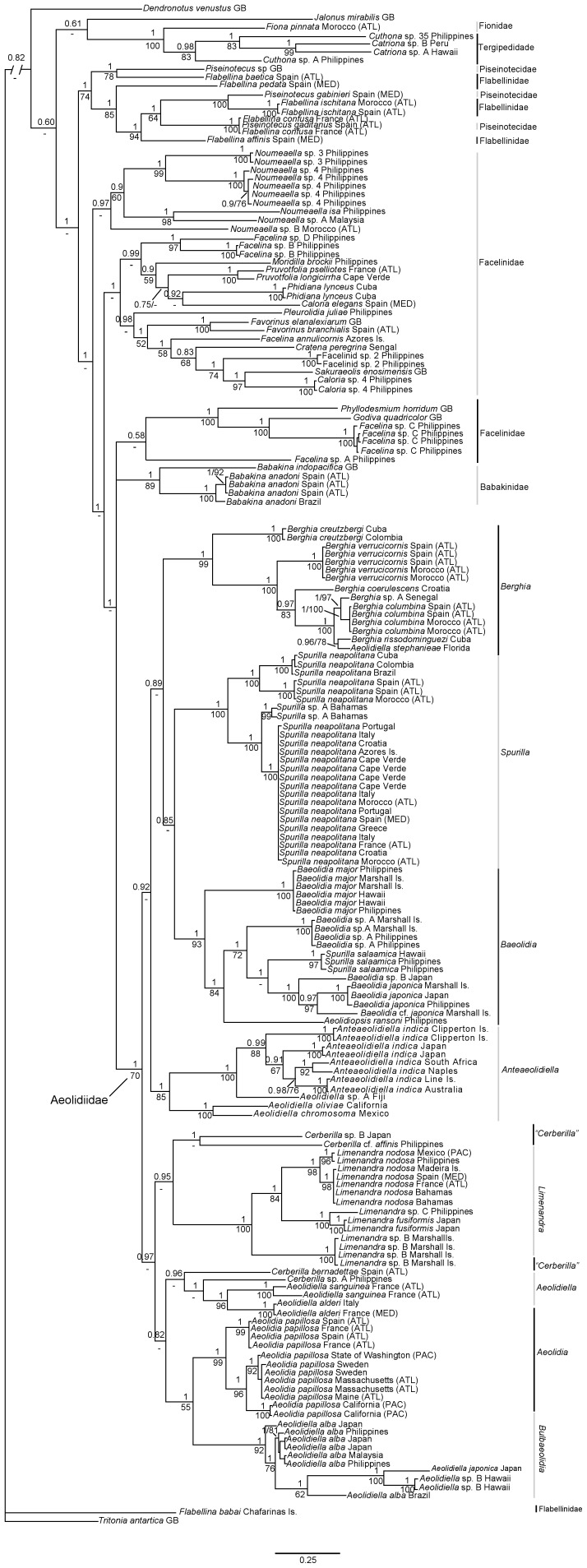
Phylogenetic hypothesis based on the combined dataset (H3+COI+16S) inferred by Bayesian analysis (BI). Numbers above branches represent posterior probabilities from BI. Numbers below branches indicate bootstrap values for ML. Abbreviations: ATL, Atlantic Ocean; EA, eastern Atlantic Ocean; GB, GenBank; MED, Mediterranean; PAC, Pacific; WA, western Atlantic Ocean. Genera names on right side of vertical bars refer to revised classification.

**Figure 2:**
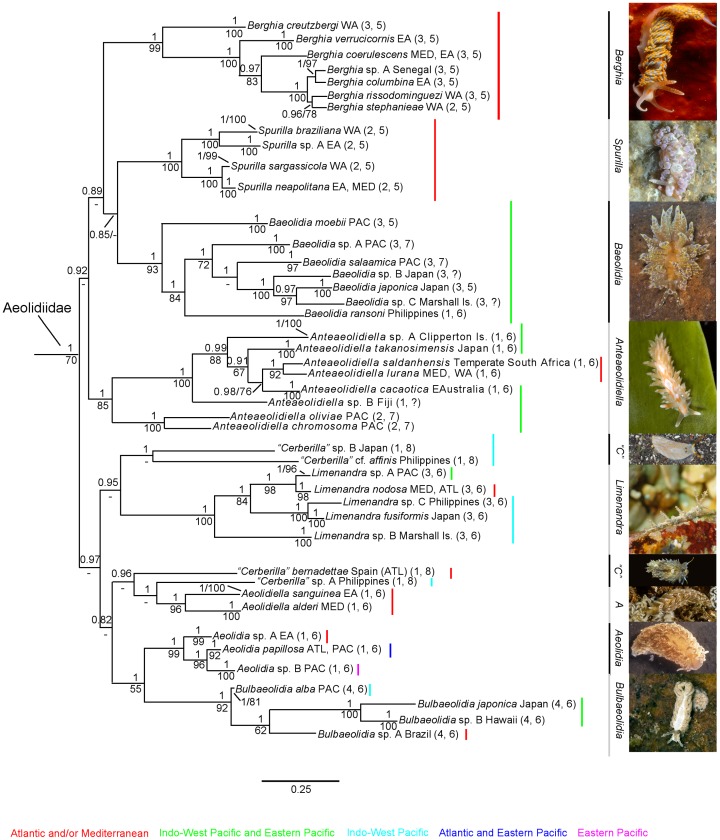
Molecular phylogeny based on the combined dataset (H3+COI+16S) inferred by Bayesian analysis (BI). Only one specimen per species of Aeolidiidae is shown. Numbers above branches represent posterior probabilities from BI. Numbers below branches indicate bootstrap values for ML. Numbers in brackets are code numbers for different states of the morphological characters listed in Table 3. Excluding *Anteaeolidiella* and *Cerberilla*, photographs depict the type species of each genus. Photo credits and details: *Berghia coerulescens*, Guido Villani, Italy; *Spurilla neapolitana*, Marina Poddubetskaia, France, Cap Ferret; *Baeolidia moebii*, Terrence M. Gosliner, Philippines; *Anteaeolidiella lurana*, Terrence M. Gosliner, Bermuda; *Cerberilla* sp. B, A. Kawahara, Japan; *Limenandra nodosa,* Marina Poddubetskaia, France, Cap Ferret; *Cerberilla* sp. A, Terrence M. Gosliner, Philippines; *Aeolidiella alderi*, Guido Villani, Italy; *Aeolidia papillosa,* Marta Pola, Sweden; *Bulbaeolidia alba,* Terrence M. Gosliner, Philippines. Abbreviations: *A*, *Aeolidiella*; ATL, Atlantic Ocean; *“C”, “Cerberilla”;* EA, eastern Atlantic Ocean; MED, Mediterranean; PAC, Pacific; WA, western Atlantic Ocean. Genera names on right side of vertical bars refer to revised classification; ?, missing data.

The combined dataset yielded a sequence alignment of 1,481positions. The ILD test showed no significant conflicting signal between the three genes (P = 0.15). No saturation was observed across genes and codon positions (not shown). The combined tree provided better resolution than H3, COI, or 16S separately (only the COI tree is provided, see Figure S1). Although bootstrap values were lower than posterior probabilities in larger clades, the topologies of the ML trees were congruent with the results yielded by Bayesian analyses. The ML tree is provided as Supplementary material (Figure S2). Figures 1 and 2 show the resulting phylogenetic hypothesis based on the combined dataset represented by BI. Figure 1 depicts species names with the preliminary identifications, whereas Figure 2 excludes the whole outgroup and shows only one representative of each species with the final revised names.

The relationship between the monophyletic Babakinidae (PP = 1, BS = 89) and some facelinid species (*Phyllodesmium horridum, Godiva quadricolor, Facelina* sp. C, and *Facelina* sp. A) with Aeolidiidae was not resolved (PP = 1, not recovered in ML). Excluding *Pleurolidia*, which clustered with some Facelinidae, Aeolidiidae was monophyletic and strongly supported in Bayesian analyses (PP = 1, BS = 70). Furthermore, Aeolidiidae was divided in two major subclades: one including *Berghia, Spurilla, Baeolidia* and *Anteaeolidiella* (PP = 0.92, not recovered in ML) and the other with *Ceberilla, Limenandra, Aeolidiella*, *Aeolidia* and an unnamed subclade (PP = 0.97, not recovered in ML). Within Aeolidiidae, and excluding *Aeolidiella, Cerberilla, Berghia, Baeolidia* and *Aeolidiopsis,* all the traditional genera included in this analysis were monophyletic and well supported by the Bayesian and Maximum likelihood analyses.

*Aeolidiella stephanieae* was the sister species of *Berghia rissodominguezi* (PP = 0.96, BS = 78; minimum uncorrected *p*-distance = 5.7% for COI), resulting in a necessary taxonomic change of the former species to preserve the monophyly of *Berghia*. In addition to the two species mentioned above, the *Berghia* clade included *B. creutzbergi, B. verrucicornis, B. coerulescens,* an undescribed species from Senegal and *B. columbina* (PP = 1, BS = 99).

*Spurilla* specimens all clustered together in a clade with the maximum support (PP = 1, BS = 100). This clade was divided into two branches. The first one was composed by western Atlantic specimens of *Spurilla neapolitana* plus a small population from western Andalusia (Spain) and Morocco (Atlantic coasts) (PP = 1, BS = 100; minimum uncorrected *p*-distance = 10.8% for COI between eastern and western Atlantic specimens). The second branch included *Spurilla* sp. A from Bahamas and the Mediterranean Sea as well as eastern Atlantic specimens of *Spurilla neapolitana* (PP = 1, BS = 100; minimum uncorrected *p*-distance = 6.4% for COI).

The Bayesian inference tree shows *Baeolidia* (PP = 1, BS = 93) as the sister group of *Spurilla*, whereas in the Maximum likelihood analyses *Baeolidia* was the sister species of *Berghia* (Figure S2). However, none of these relationships were supported (PP = 85, not recovered in ML). *Aeolidiopsis ransoni* and *Spurilla salaamica* clustered among *Baeolidia* species. *Baeolidia major* specimens clustered in a single subclade (PP = 1, BS = 84). The other subclade was composed by an undescribed species from the Philippines and the Marshall Is., *Spurilla salaamica*, an undescribed species from Japan, *Baeolidia japonica* from Marshall Is., Japan and the Philippines and one specimen of *Baeolidia* cf. *japonica* from Marshall Is. (minimum uncorrected *p*-distance = 12.6% for COI between this specimen and *B. japonica*). *Aeolidiopsis ransoni* was basal to the latter subclade (PP = 1, BS = 84).

*Anteaeolidiella* was the sister group of *Berghia, Spurilla* and *Baeolidia* (PP = 0.92, not recovered in ML), appearing as a well-supported lineage (PP = 1, BS = 85). *Anteaeolidiella* was split into two groups: *Aeolidiella chromosoma* and *Aeolidiella oliviae* formed the first and basal clade (PP = 1, BS = 100), whereas an undescribed species from Fiji together with *Anteaeolidiella indica* specimens constituted the second clade (PP = 1, BS = 100). These specimens previously identified as *A. indica* were divided into four different subclades (PP = 1, BS = 88; minimum uncorrected *p*-distance = 8.4% for COI among clades) and likely include five distinct species.

*Cerberilla* was not recovered as monophyletic. *Cerberilla affinis* and *Cerberilla* sp. B from Japan formed the sister group of *Limenandra* (PP = 0.95, not recovered in ML). However, *Cerberilla bernadettae* and *Cerberilla* sp. A from the Philippines clustered in a separate clade basal to some *Aeolidiella* species (PP = 0.96, not recovered in ML).

*Limenandra* specimens clustered in a clade with maximum support (PP = 1, BS = 100). This clade was divided into two subgroups. All *Limenandra nodosa* specimens formed a monophyletic group with maximum support (PP = 1, BS = 98), subdivided in Pacific and Mediterranean-Atlantic clusters (minimum uncorrected *p*-distance = 7.3% for COI between Pacific and Mediterranean-Atlantic specimens). The maximum genetic distance between eastern and western Atlantic population was 1%. *Limenandra fusiformis* and an undescribed species from the Philippines (PP = 1, BS = 100) appeared as the sister group of “*L. nodosa*” complex (PP = 1, BS = 84). Another undescribed species from the Marshall Islands completed this clade, forming the second subgroup that is sister to the remainder of *Limenandra*.

Specimens previously ascribed to *Aeolidiella* clustered in two distinct and separated clades with maximum support, rendering this taxon polyphyletic. *Aeolidiella alderi* (type species) clustered with *Aeolidiella sanguinea* (PP = 1, BS = 96), whereas *Aeolidiella alba, Aeolidiella japonica*, an undescribed species from Hawaii and one specimen of *Aeolidiella alba* from Brazil formed the second clade (PP = 1, BS = 92). These latter taxa formed the sister group to *Aeolidia* (PP = 1, not recovered in ML). The basal position of one of the specimens of *A. alba* from Japan can be explained because its 16S sequence presented some insertions. Genetic distance between *Aeolidiella alba* from the Indo-Pacific and from the western Atlantic was 13.5% (minimum uncorrected *p*-distances for COI).

Finally, specimens of *Aeolidia papillosa* were placed in two different clades both with maximum support. The first clade included specimens from the Atlantic coast of France and from Galicia (Spain). The second clade was further divided into two subclades: specimens from Sweden, one specimen from Maine (USA), two from Massachusetts (USA) and one specimen from the state of Washington (USA) clustered together; the second subclade included two specimens from the California coast. The minimum *p*-distance among all *Aeolidia* clades was 8.4% (uncorrected *p*-distances for COI).

Flabellinidae, Facelinidae and Piseinotecidae all appeared to be paraphyletic or polyphyletic. Fionidae appeared as the sister group of Tergipedidae (PP = 1, BS = 100).

## Discussion

### Aeolidiidae

Our results support Aeolidiidae as a monophyletic family, as long as *Pleurolidia juliae* is excluded. This contrasts with the results obtained by Carmona et al. [13], who stated that Aeolidiidae was not monophyletic due to *Facelina punctata* (Alder & Hancock, 1845) -a junior synonym of *F. annulicornis* (Chamisso & Eysenhardt, 1821)- clustered among the aeolidiid species included in their study. The *Facelina punctata* COI sequence used in the above paper was taken from GenBank [16]. In order to avoid the possibility of a misidentification of such facelinid, in the present study, we sequenced mitochondrial (COI and 16S) and nuclear (H3) genes from a *Facelina punctata* specimen collected from the Azores Is. and identified by the authors. The inclusion of these sequences in our analyses did not affect the monophyly of Aeolidiidae. This suggests caution when using the specimen of *Facelina punctata* from GenBank in analysing the phylogenetic relationships of this group.

The close relationship among Aeolidiidae, Babakinidae and a clade composed by several Facelinidae, suggested in previous studies [10], [13], was confirmed using a much broader taxon sampling.

The monophyly of *Aeolidia*, *Anteaeolidiella*, *Limenandra* and *Spurilla* was highly supported. Intergeneric relationships were also well supported in the Bayesian analyses but not in the Maximum likelihood analyses. Neither Aeolidiidae and Spurillidae *sensu* Odhner [29] and Odhner (in [37]), nor Spurillinae or Aeolidiinae *sensu* Schmekel & Portmann [42] were recovered as monophyletic taxa Therefore, in order to minimize disruption of the nomenclature and to choose the most parsimonious option, the validity of these taxa is rejected.

### *Pleurolidia*

In our study, *Pleurolidia* clusters with some facelinids, but not within the traditional Aeolidiidae. *Protaeolidiella* and *Pleurolidia* are two monospecific genera that feed on hydroids instead of anemones. They have been considered as primitive aeolidiids [4], [75]–[76]. Burn [76] described *Pleurolidia juliae* based on two specimens, although most of the information was referred to the holotype, which possesses a triseriate radula. He pointed out that no details of the buccal bulb of the second specimen were known due to the fact that it was lost. Burn [76] also mentioned that the holotype and the second specimen of his species might not be conspecific. *P. juliae* would be the only aeolidiid with a triseriate radula because *Protaeolidiella atra* has an uniseriate radula [77]. But, since no traces of the lateral teeth of *Pleurolidia juliae* radula were found, Rudman [44] concluded that both were conspecific and therefore rendered *P. juliae* as junior synonym of *Protaeolidiella atra*. Two years later, Baba [78] stated that both species were valid although he did not examine the radula of *Pleurolidia juliae.* Here, we re-examined material of *P. juliae* and found no trace of lateral teeth (not shown). Hence, there are three possible hypotheses for the triseriate radula of *P. juliae*: intraspecific variation; an aberrant radula, which has been observed in other Aeolidida species [9], [79] or; a misidentification of this specimen with another aeolid that has a triseriate radula. *P. juliae*’s diet matches that of most facelinds’ (hydroids rather than sea anemones). This fact further supports the molecular results we obtained where *P. juliae* is included in Facelinidae. Inclusion of *Protaeolidiella atra* specimens in future analyses would be worth in order to test this apparent relationship and distinctness in relation to *Pleurolidia juliae*.

### *Berghia*

The monophyly of the genus *Berghia* was highly supported when *Aeolidiella stephanieae* was included. *Berghia* is entirely restricted to the Atlantic and interestingly, none of the species studied here have an amphiatlantic distribution. This genus has been considered a junior synonym of *Spurilla* by some authors [30], [80]. Nevertheless, our results show that *Berghia* is not as closely related to *Spurilla,* as previously thought. In our analysis, this genus includes *B. creutzbergi, B. verrucicornis, B. coerulescens,* an undescribed species from Senegal, *B. columbina*, *B. rissodominguezi* and *Aeolidiella stephanieae*. Concerning the latter species, Valdés [81] placed it within *Aeolidiella* following the diagnosis of Gosliner [4]. This species, which is commercially important as a biological control of *Aiptasia* infestations in aquaria [46], [81]–[83], has been misidentified as the Mediterranean species *Berghia verrucicornis* (see [31], [82]–[84]) and *Berghia coerulescens* [46] for many years. However, *A. stephanieae* clusters together with *Berghia rissodomiguezi*, which also has been suggested by Valdés et al. [46] as a possible junior synonym of *Berghia coerulescens.* Both species, *B. rissodominguezi* and *A. stephanieae,* have differences in coloration, ceratal arrangement, rhinophorial ornamentation, genital and anal apertures as well as the masticatory border and the central cusp denticle [81], [85]. Therefore, although the uncorrected *p*-distance between both specimens is at the lower limit (5.7%) of our range for sister species, we concluded that those morphological differences are significant enough to consider both species as distinct. Hence, *Aeolidiella stephanieae* should be renamed as *Berghia stephanieae*.

### *Spurilla*

*Spurilla* is monophyletic with maximum support. Its type species, *Spurilla neapolitana*, has been intensively studied by several authors [41]–[42], [86]–[95]. According to the literature, the intraspecific variation of the denticulation of the jaws, the shape of the central cusp and its coloration are widely accepted. Furthermore, *Spurilla neapolitana* has been considered a circumtropical species (e.g. [41]). Nevertheless, our results clearly demonstrate the existence of a complex of at least three cryptic species under the name *S. neapolitana* and reject its amphiatlantic or circumtropical distribution. The genetic distance between eastern and western Atlantic specimens attributed to this species (minimum uncorrected *p*-distance = 14.1%) is at the upper limit of the range here considered for different species. Hence, the true *Spurilla neapolitana* is distributed along the Mediterranean Sea as well as the eastern Atlantic Ocean (from Portugal to Cape Verde, including the Azores Is.). Regarding the western Atlantic population, MacFarland [96] erected the name of *Spurilla braziliana* for the Brazilian specimens of this genus. This species has been considered as a junior synonym of *S. neapolitana* [33], [42]–[43], [46], [95], [97]–[99]. The present study clearly demonstrates that both names correspond to valid species and highlights the difficulties of identifying these species without a molecular phylogenetic framework. Two specimens from Japan and two specimens from the Pacific coast of Costa Rica clustered together with the western Atlantic specimens (Figure S1), showing a 1.9% difference in their COI gene among them. These specimens do not appear in Figure 1 because sequencing of 16S and H3 failed (see Table S1). This outcome suggests that those *“S. neapolitana”* reported from the Pacific Ocean [41], [100]–[103] may be *S. braziliana.*

A second sibling species of *S. neapolitana* was detected from specimens from Huelva (SW Spain, Atlantic Ocean) and Agadir (Morocco, Atlantic Ocean). Considering its geographical distribution, this second species could be ascribed to *Spurilla mograbina* or *Spurilla dakariensis.* Pruvot-Fol [104] described *Spurilla mograbina* and *Spurilla dakariensis* from material from Temara (Morocco) and Dakar (Senegal) respectively. However, it is not possible to positively identify the specimens from Agadir and Huelva as *S. mograbina.* Pruvot-Fol’s figure (pl. 1, fig. 12) [104] depicts a single *Spurilla* specimen with opaque cream spots scattered along the dorsal side of the cerata and the back, especially behind the pericardium, but there are no traces of these cream spots in our specimens. Due to the fact that Pruvot-Fol classified *S. mograbina* as a variety of *S. neapolitana* and some of our specimens from Agadir and Temara cluster within the clade of the true *S. neapolitana, S. mograbina* is probably a junior synonym of *S. neapolitana*.

The original description of *Spurilla dakariensis* [104] is very brief and vague. With the scarce information provided by Pruvot-Fol [104] some colour types of *Spurilla neapolitana*, *Spurilla* sp. A and *Spurilla braziliana* could be attributed as *Spurilla dakariensis*. Thus, since this name is unidentifiable, we conclude that *S. dakariensis* should be treated as *nomen dubium*.

Finally, our results show that the sister species of *S. neapolitana* is *S. sargassicola* (Kröyer in Bergh, 1861). This latter species has been considered a junior synonym of *S. neapolitana* by most authors [33], [93], [95], [99]. Recently, Valdés et al. [46] defended its validity by comparing a specimen of each species in the same photograph (see p. 270). Based on differences in coloration, ceratal arrangement and our molecular data, we confirm that both names correspond to closely related, but separate species.

### *Baeolidia*

*Baeolidia moebii* Bergh, 1888, the type species of this genus, supposedly has not been found since it was described from the Mauritius Is. When Eliot [105] described *Baeolidia major* from Zanzibar, he stated that the main difference between both species was their size (8 mm long for *B. moebii* and 40 mm for *B. major*). Even though he pointed out that *B. major* could be merely a full-grown individual of *B. moebii,* Eliot [105] finally considered *B. major* as a valid species. Besides habitat similarities [105], both species also share some morphological features such us colouration, the leaf-like cerata and the radular morphology. Hence, we suggest that both names are conspecific and consider *Baeolidia major* to be a junior synonym of *Baeolidia moebii*.

The monophyly of *Baeolidia* is well supported only when *Aeolidiopsis ransoni* and *Spurilla salaamica* are included in *Baeolidia*. Since *Aeolidiopsis* has never been considered as a junior synonym of Baeolidia, the placement of *Aeolidiopsis ransoni* within *Baeolidia* represents an intriguing outcome. *Aeolidiopsis ransoni* has been retained as the sole species of *Aeolidiopsis* due to its acleioproctic anus dorsal to the notal brim and its smooth rhinophores [4], [8], [106]. Our results suggest that the position of the anus and the ornamentation of the rhinophores cannot be diagnostic for Aeolidiidae genera (see *Cerberilla* discussion). Therefore, we suggest transferring *Aeolidiopsis ransoni* to *Baeolidia* that includes the other two species that feed on the zooanthid *Palythoa, Baeolidia harrietae* (Rudman, 1982) and *Baeolidia palythoae* Gosliner, 1985 [4]. Further studies including molecular analyses with *B. harrietae* and *B. palythoae* are needed to clarify the relationships among these species.

Since Rudman [30] considered *Berghia* and *Baeolidia* as junior synonyms of *Spurilla* and despite its papillate rhinophores, he ascribed *Spurilla salaamica* to *Spurilla*. Gosliner [4] transferred this species to *Berghia* based on its papillate rhinophores and cerata in arches. Despite the traditional use of ceratal arrangement and rhinophorial ornamentation to separate genera, the present study highlights that these characters cannot be diagnostic for most aeolidiid genera (see the last section of the discussion). Hence, the most parsimonious alternative is to rename *Spurilla salaamica* as *Baeolidia salaamica* although further morphological studies are needed in order to find synapomorphies. Members of this lineage are restricted to the Indo-Pacific tropics and the eastern Pacific.

### *Anteaeolidiella*

Our results confirmed the validity of the recent genus *Anteaeolidiella* [8] from a molecular point of view. The type species, *Anteaeolidiella indica* (Bergh, 1888), has been considered to have a worldwide distribution [2]–[3], [10], [46]. Several synonyms have been attributed to this species: *Aeolis foulisi* Angas, 1864, *Aeolidiella orientalis* Bergh, 1888, *A. saldanhensis* Barnard, 1927, *A. hulli* Risbec, 1928, *A. takanosimensis* Baba, 1930, *A. multicolor* Macnae, 1954 and *A. lurana* Marcus & Marcus, 1967 [3], [107]. However, our study demonstrates that at least five distinct allopatric species constitute the *Anteaeolidiella indica* complex, which presents to a consistent biogeographic pattern. In 1855, Stimpson [108] described *Eolis cacaotica* from Port Jackson (Sydney Harbour, eastern Australia), while Burn [109] recently suggested that this could be the older name of *Aeolis foulisi*, which was also described from Port Jackson. Upon examining the original descriptions of these species, we concluded that both descriptions refer to the same taxon and concur with our specimens from Australia. Therefore, we consider *Aeolis foulisi* as a junior synonym of *Eolis cacaotica* and transfer the latter to *Anteaeolidiella*. A Japanese specimen that shares the colouration pattern of *A. cacaotica* (not shown) clusters among the Australian specimens of *A. cacaotica*. This outcome suggests that this species is also present in Japan. The Japanese specimen does not appear in Figure 1 because sequencing of COI and H3 failed (see Table S1).

Regarding our specimen from South Africa, Bergh [110] described *Aeolidiella indica* from the Mauritius Is. as “whitish-yellow or greyish-yellow body; grey rhinophores with white tips; white tentacles with yellow tips and grey or yellow cerata”. Later, Barnard [111] erected the South African species *Aeolidiella saldanhensis* from a preserved specimen and only provided information about the radula and foot shape. Macnae [112] described *Aeolidiella multicolor* with material from South Africa based on differences in coloration, size and radular teeth with *A. indica* and differences in foot corners shape with *A. saldanhensis*. Nevertheless, Macnae pointed out that the coloration of this species was very variable. Indeed, the most remarkable characteristic of this *Aeolidiella*, the bluish background of the cerata, presents high intraspecific variation. Just considering coloration, Bergh’s species matches the tropical South African “*Aeolidiella indica*” specimens, whereas our South African specimen from temperate waters is concurrent with *A. multicolor* (not shown). Hence, we think that both names are non-conspecific but we treat *A. multicolor* as a junior synonym of *A. saldanhensis* and conclude that our specimen from temperate South Africa belongs to this last species. A redefinition of *A. indica* is needed in order to clarify the morphological characteristics of this species and its intraspecific variability.

The coloration of our Japanese specimens matches with the original drawings of *Anteaeolidiella takanosimensis* [113]. Although *A. takanosimensis* is reported as a cosmopolitan species [3], our data suggest that this species is restricted to Japan.

Genetic distance, as well as external differences (not shown), suggest that “*Anteaeolidiella indica*” from Naples (Mediterranean Sea) is *Anteaeolidiella lurana*. This species was described based on material from Brazil by Marcus & Marcus in 1967 [93]. However, in our analyses one specimen from Brazil and another from Bermuda clustered together with the specimen from Naples (maximum uncorrected *p*-distance = 0%). The Brazilian and Bermudan specimens do not appear in Figure 1 because sequencing of some of the genes failed (see Table S1 and Figure S1). Therefore, *Anteaeolidiella lurana* seems to be an amphiatlantic species closely related to *A. saldanhensis,* differing from this species molecularly and in their rhinophores and oral tentacles’ colour pattern.

We could not determine the identity of those specimens from the Clipperton Island. They present differences in body shape and coloration with respect to the rest of *Anteaeolidiella* species, including *A. chromosoma* and *A. oliviae.* The eastern Pacific taxon most likely represents an undescribed species, based on its genetic distance from other members of this clade (minimum uncorrected *p*-distance = 14.2%).

### *Cerberilla*

*Cerberilla* is the only aeolidiid genus whose systematics remains unresolved. Because of their burrowing behaviour, not many specimens of *Cerberilla* [79]–[80], [114] are encountered and very few are properly preserved for molecular work. For this reason only seven species have been included in our analyses (Table S1).

The marginal denticle, the occurrence of small denticles between larger denticles of the radula and its pleuroproctic anus have traditionally distinguished this genus from the rest of aeolidiids [4], [8], [76], [115]. Due to its pleuroproctic anus, species of *Cerberilla* have been considered as primitive aeolidiids together with *Protaeolidiella* and *Pleurolidia* [4], [75]–[76]. Nevertheless, our analyses clearly showed that the position of the anus does not have any phylogenetic significance within Aeolidiidae (e.g. *Aeolidiopsis ransoni,* here transferred to *Baeolidia*). Tardy [75] pointed out that a classification based on the position of the anus “leads to the formation of tribes that may often appear to be artificial in the light of new investigations”. Furthermore, within each “*Cerberilla*” clade, a huge intrageneric variability of the radula can be observed. A “classical” *Cerberilla* radula (e.g. *Cerberilla affinis*) clusters together with a “classical” *Aeolidiella* radula (e.g. *Ceberilla* sp. B from Japan).

A more comprehensive study including the type species of the genus (*Cerberilla longicirrha* Bergh, 1873), more *Cerberilla* species and a detailed examination of their morphology is certainly required in other to clarify its phylogenetic status.

### *Limenandra*

Many authors have considered *Limenandra* as a junior synonym of *Baeolidia* [4], [41], [46]–[47]. Nevertheless, our study confirmed the validity of this genus and its monophyly. *Baeolidia* and *Limenandra* are not even sister groups. Rather, *Limenandra* is more closely related to some “*Cerberilla”* species. *Limenandra nodosa*, the type species of the genus, is amphiatlantic but is not so widely distributed as it is referred to in the bibliography [4], [41], [46]–[47]. Specimens from the Pacific (the Philippines and Mexico) attributed to *Limenandra nodosa* appear to be a distinct species that is sister to *L. nodosa* (the Atlantic and the Mediterranean) (minimum uncorrected *p*-distance = 7.3% between both species).

On the other hand, the sister group of the above clade is composed of *L. fusiformis* and an undescribed species, *Limenandra* sp. C (*Baeolidia* sp. 3 in [47]), from the Philippines. All these taxa form a clade that is the sister taxon to *Limenandra* sp. B (*Baeolidia* sp. 2 in [47]), from the Marshall Islands and the Philippines. Therefore, *Limenandra* is clearly far more diverse than previous studies have indicated.

### *Aeolidiella*

*Aeolidiella* is the aeolidiids genus to which most nominal species have been ascribed (see Table S3). Nevertheless, our data depict a completely different scenario. *Aeolidiella alderi* (type species) and *A. sanguinea* constitute a small and monophyletic group included in a larger clade that includes some species of “*Cerberilla”*. The remaining *Aeolidiella* species included in this study (*Aeolidiella alba*, *Aeolidiella japonica*, *Aeolidiella* sp. B and the Brazilian specimens attributed to *Aeolidiella alba)* cluster together as the sister group of *Aeolidia*. Therefore, the traditional *Aeolidiella* is rendered as polyphyletic. In order to recover its monophyly, a new name for the clade constituted by *A. alba*, *A.japonica*, *Aeolidiella* sp. B and the specimens attributed to *Aeolidiella alba* from Brazil is erected. The relationship of *Aeolidiella* with “*Ceberilla” bernadettae* and “*Ceberilla”* sp. A still needs further study. So far, the genus *Aeolidiella* is restricted to the Atlantic-Mediterranean.

### *Aeolidia*

*Aeolidia* is the type genus of the family and the sister group of a clade named below and composed of several species previously attributed to *Aeolidiella*. The monophyly of *Aeolidia* is highly supported. Our results show the existence of a complex of three sibling species under the name of *A. papillosa* besides confirming the amphiatlantic status of this species. Thus, the true *A. papillosa* is distributed from Sweden (close to the type locality) to the Northeastern Pacific coasts (state of Washington), including the western Atlantic (maximum uncorrected *p*-distance = 1.6%). Its sister taxon is a sibling species from California (minimum uncorrected *p*-distance = 8.4% between both clades). A third cryptic species includes specimens early attributed to “*Aeolidia papillosa*” from Cap Ferret (France, Atlantic) and Galicia (NW Spain, Atlantic) (minimum uncorrected *p*-distance = 11% between this clade and the true *A. papillosa* clade). Other *Aeolidia* species, such as *A. herculea* Bergh, 1894 and *A. collaris* Odhner, 1921 were not included in this study as material properly preserved for molecular purposes was not available.

### *Bulbaeolidia* gen. nov

LSID urn:lsid:zoobank.org:act:EA7635CA-F380-4BED-9663-298641A2C1B8.

*Aeolidiella alba* and *Aeolidiella japonica* were originally ascribed to the genus *Aeolidiella* by Risbec [116] and Eliot [117] respectively. However, in our study these species constitute a monophyletic and high supported group (PP = 1, BS = 92) together with an undescribed species from Hawaii and one specimen attributed to *A. alba* from Brazil. This outcome renders the traditional *Aeolidiella* polyphyletic. On the other hand, this clade should not be joined with *Aeolidia,* its sister clade, (Figures 1 and 2) due to the anatomical differences as well as these “*Aeolidiella*” species have never been ascribed to *Aeolidia.* Hence, in order to choose the most parsimonious option *“A”. alba*, *“A”. japonica*, *“Aeolidiella”* sp. B and the Brazilian *“Aeolidiella alba”* were grouped in the new genus *Bulbaeolidia* gen. nov. This genus is characterized by a low, wide body, with an inconspicuous pericardial swelling. Head large with short oral tentacles that are constricted at the midpoint. Rhinophores with a pair of prominent bulbous swellings. Cerata club-shaped, in single rows, with the number decreasing posteriorly and slightly curved inwards towards dorsal mid line. Cleioproctic, reproductive aperture immediately below the second ceratal row. Oral glands, composite, moderately large, tubular and tapering. Radular tooth bilobed and deeply indented, with a prominent central cusp. Jaw masticatory process smooth. The new name combines the bulbous swellings in the rhinophores of the species in the clade with the name of its sister group, *Aeolidia.*

Concerning the cryptic species from Brazil previously attributed to *Aeolidiella alba*, more Atlantic specimens from Florida, as well as deep examination of their morphology, are needed in order to clarify its geographical distribution.

### Are Traditional Morphological Characters Useful for Phylogenetic Inferences in Aeolidiidae?

Traditionally, the position of the anus, the shape of the radular tooth, the ceratal arrangement and the ornamentation of the rhinophores have been the most important characters within Aeolidiidae [4], [8], [30], [118]. However, there is little consensus regarding which of these characters are most systematically and phylogenetically informative. Indeed, depending on the author the emphasis on each morphological character varies. These disagreements have questioned the validity of some aeolidiid genera and the addition of new species has blurred the differences between these genera, especially with regard to species placed in *Spurilla, Berghia, Limenandra* and *Baeolidia*.

Our results clearly show that none of these characters have any unique phylogenetic signal within Aeolidiidae (see *Berghia, Baeolidia* and *Cerberilla* discussion). In order to illustrate these results, Table 3 shows the different states of ceratal arrangement and the rhinophoral ornamentation in Aeolidiidae while Figure 2 maps their evolution within this family. Thus, we can conclude that ornamented rhinophores and cerata in rows or/and arches have evolved independently within different lineages. For instance, considering smooth rhinophores as the plesiomorphic state [6], papillate rhinophores have evolved separately three times in Aeolidiidae. Recent molecular analyses highlight the need to re-examining the morphological characters according to relationships revealed *a posteriori* [119]–[124]. The finding of new morphological synapomorphies requires additional studies and is beyond the scope of this study.

**Table 3:** Code numbers for different states of the morphological characters traditionally used in Aeolidiidae.

Code	Morphological character
1	Smooth rhinophores
2	Perfoliate rhinophores
3	Papillate rhinophores
4	Rhinophores with a pair of prominent bulbous swellings
5	Cerata in arches
6	Cerata in rows
7	Cerata in arches and rows
8	Cerata on elevated cushions

### Conclusions

This study includes only a small representation of the aeolid heterobranchs diversity, but triplicates the number of species and quintuplicates the number of specimens included by Carmona et al. [13], the largest aeolid dataset analysed to date. Together with Johnson & Gosliner [125], this study constitutes the most extensive molecular data set for a group of heterobranchs and includes three molecular markers (COI, 16S rRNA and histone H3). Table S3 lists the ninety-seven described Aeolidiidae species. Our study includes the fifty per cent of the known species. Although our study covers only half of them, lack of material properly preserved for genetic research makes it presently impossible to undertake a more comprehensive molecular phylogenetic study of this clade. Moreover, many species such as *Milleraeolidia ritmica, Burnaia helicochorda, Baeolidia cryoporos* and *Berghia chaka* have not been reported since they were originally described. Likewise, many old taxa were poorly described and it will be a challenge to recognize such taxa even if they were encountered again. In conclusion, this study confirms the monophyly of Aeolidiidae, excluding *Pleurolidia*. Neither Spurillidae/Spurillinae nor Aeolidiinae were recovered as monophyletic taxa. After some changes in the systematics of the family here proposed, Aeolidiidae includes eight monophyletic genera: *Berghia, Spurilla, Baeolidia, Anteaeolidiella, Limenandra, Aeolidiella, Aeolidia* and *Bulbaeolidia* gen. nov. The taxonomic status of *Cerberilla,* however, remains unresolved. Our results also suggest the existence of four sibling-species complexes within Aeolidiidae, which may increase to a total of 115 species, including 18 undescribed species and the resurrection of six species previously in synonymy. Finally, this contribution highlights the necessity of additional research to identify and characterize new morphological synapomorphies to better explain the phylogenetic relationships within this family.

## Supporting Information

Figure S1 **Molecular phylogeny inferred from partial sequences of the individual mitochondrial COI gene by Bayesian analysis.** Numbers above branches represent posterior probabilities from BI. Numbers below branches indicate bootstrap values for ML. Abbreviations: ATL, Atlantic Ocean; EA, eastern Atlantic Ocean; GB, GenBank; MED, Mediterranean; PAC, Pacific; WA, western Atlantic Ocean.
(TIF)

Figure S2 **Phylogenetic hypothesis based on the combined dataset (H3+COI+16S) inferred by Maximum likelihood analysis (ML).** Numbers above branches indicate bootstrap values for ML. Abbreviations: ATL, Atlantic Ocean; EA, eastern Atlantic Ocean; GB, GenBank; MED, Mediterranean; PAC, Pacific; WA, western Atlantic Ocean. Genera names on right side of vertical bars refer to revised classification.
(TIF)

Table S1 **List of specimens used for phylogenetic analyses.** We include both the species names resulting from our morpho-chromatic identification (provisional ids) and the names after analyses (final ids; this only when changes have occurred). EA = eastern Atlantic Ocean; EP = eastern Pacific; GB = GenBank; MED = Mediterranean; WA = western Atlantic Ocean.
(DOCX)

Table S2 **Minimum COI gene pairwise uncorrected** ***p*** **-distances between sister species of each genus.**
(DOCX)

Table S3 **List of nominal species of Aeolidiidae.** Final identifications are determined after a review of the pertinent literature. ✓ = species analysed here; X = species not found properly preserved for molecular analyses; − = validity and/or identity of this species not studied here.
(DOCX)
