# Supplementary figures and images for: A Tale That Morphology Fails to Tell: A Molecular Phylogeny of Aeolidiidae (Aeolidida, Nudibranchia, Gastropoda)
Source: PLoS One. 2013 May 2;8(5):e63000. doi: 10.1371/journal.pone.0063000 (PMC3642091; doi:10.1371/journal.pone.0063000)

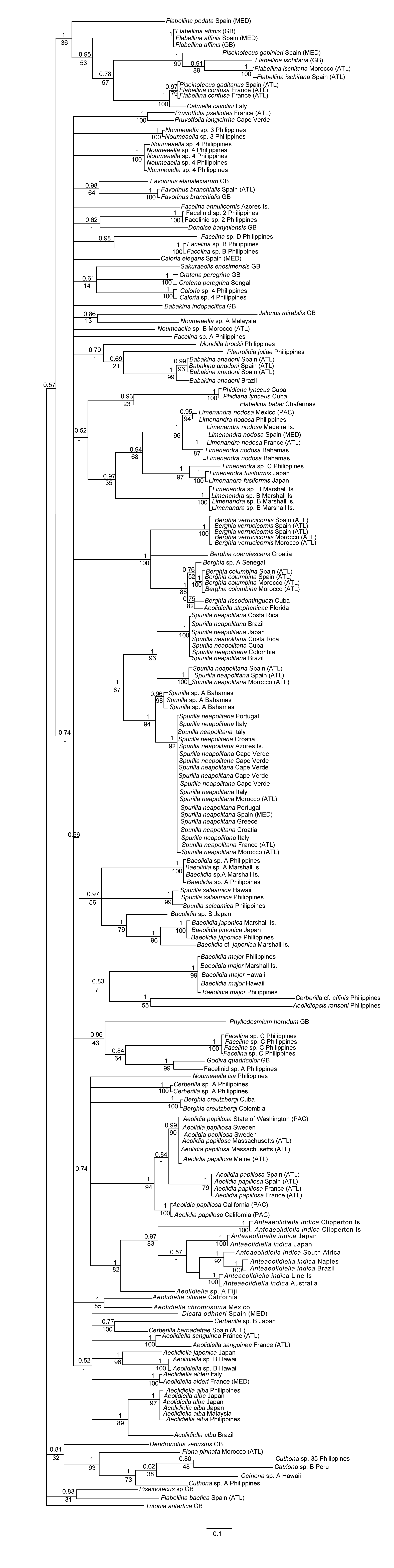

Supplement: Figure S1 — Molecular phylogeny inferred from partial sequences of the individual mitochondrial COI gene by Bayesian analysis. Numbers above branches represent posterior probabilities from BI. Numbers below branches indicate bootstrap values for ML. Abbreviations: ATL, Atlantic Ocean; EA, eastern Atlantic Ocean; GB, GenBank; MED, Mediterranean; PAC, Pacific; WA, western Atlantic Ocean. (TIF) [file pone.0063000.s001.tif]

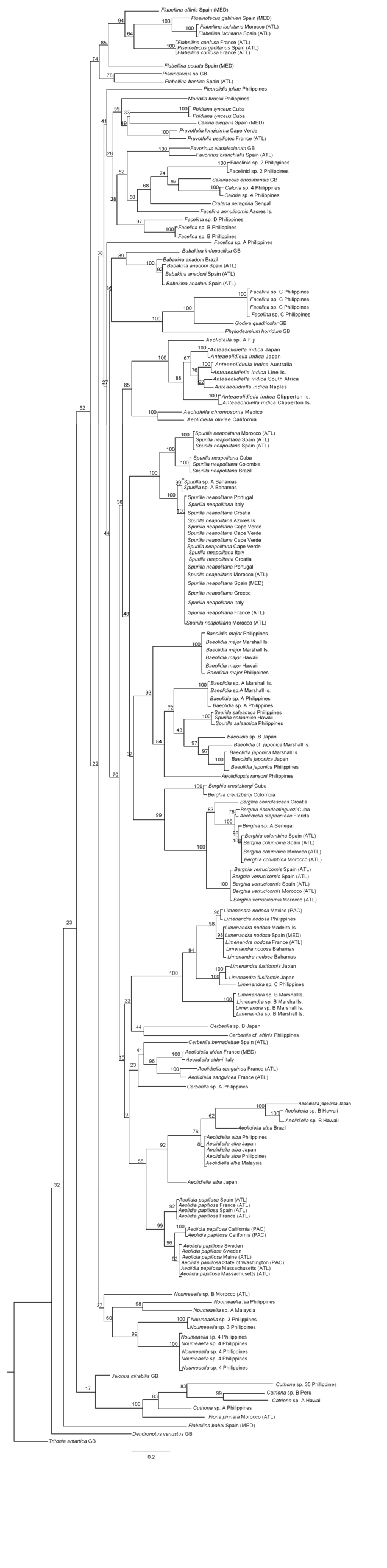

Supplement: Figure S2 — Phylogenetic hypothesis based on the combined dataset (H3+COI+16S) inferred by Maximum likelihood analysis (ML). Numbers above branches indicate bootstrap values for ML. Abbreviations: ATL, Atlantic Ocean; EA, eastern Atlantic Ocean; GB, GenBank; MED, Mediterranean; PAC, Pacific; WA, western Atlantic Ocean. Genera names on right side of vertical bars refer to revised classification. (TIF) [file pone.0063000.s002.tif]
