# Supplementary material for: A Tale That Morphology Fails to Tell: A Molecular Phylogeny of Aeolidiidae (Aeolidida, Nudibranchia, Gastropoda)
Source: PLoS One. 2013 May 2;8(5):e63000. doi: 10.1371/journal.pone.0063000 (PMC3642091; doi:10.1371/journal.pone.0063000)
Supplement: Table S1 — List of specimens used for phylogenetic analyses. We include both the species names resulting from our morpho-chromatic identification (provisional ids) and the names after analyses (final ids; this only when changes have occurred). EA = eastern Atlantic Ocean; EP = eastern Pacific; GB = GenBank; MED = Mediterranean; WA = western Atlantic Ocean. (DOCX) [file pone.0063000.s003.docx]

| **Family** | **Species** | | **Locality** | **Collection dates** | **Voucher** | **GenBank Accession Nos.** | | |
| --- | --- | --- | --- | --- | --- | --- | --- | --- |
|  | **Preliminary ids** | **Revised ids** |  |  |  | **COI** | **16S** | **H3** |
| Tritoniidae Lamarck, 1809 | *Tritonia antarctica* Pfeffer, 1886 |  | Bouvetoya (EA, GB) | 30Jun04 |  | HM162718 | HM162643 | HM162550 |
| Dendronotidae Allman, 1845 | *Dendronotus venustus* MacFarland, 1966 |  | Santa Monica (California, GB) | Dec07 |  | HM162709 | HM162630 | HM162536 |
| Proctonotidae Gray, 1853 | *Janolus mirabilis* Baba & Abe, 1970 |  | Philippines (GB) | 19May09 |  | HM162750 | HM162674 | HM162583 |
| Aeolidiidae Gray, 1827 | *Aeolidia papillosa* (Linnaeus, 1761) | *Aeolidia* sp. B | California | 26May01 | CASIZ 184504 | JQ997035 | JQ996830 | JQ996931 |
|  |  | *Aeolidia* sp. B | California | 28Jan10 | CASIZ 182214 | JQ997036 | JQ996831 | JQ996932 |
|  |  | *Aeolidia* sp. A | France (EA) | 01Jun09 | MNCN/ADN: 51926 | JX087531 | JX087459 | JX087593 |
|  |  | *Aeolidia* sp. A | France (EA) | 18Jun09 | MNCN/ADN: 51927 | JX087532 | JX087460 | JX087594 |
|  |  |  | Maine | 09Aug09 | CASIZ 182329 | JQ997038 | JQ996833 | JQ996934 |
|  |  |  | Massachusetts | Nov10 | CASIZ 187760 | JQ997039 | JQ996834 | JQ996935 |
|  |  |  | Massachusetts | Nov10 | CASIZ 187760 | JQ997040 | JQ996835 | JQ996936 |
|  |  |  | Massachusetts | 26Oct11 | CASIZ 187841 | JQ997041 | JQ996836 | - |
|  |  |  | Massachusetts | 26Oct11 | CASIZ 187841 | JQ997042 | - | JQ996937 |
|  |  | *Aeolidia* sp. A | Spain (EA) | 17Sept11 | MNCN/ADN: 51928 | JX087533 | JX087461 | JX087595 |
|  |  | *Aeolidia* sp. A | Spain (EA) | 17Mar11 | CASIZ 187742 | JQ997037 | JQ996832 | JQ996933 |
|  |  |  | Sweden | 14Sep11 | MNCN/ADN: 51929 | JX087534 | JX087462 | JX087596 |
|  |  |  | Sweden | 14Sep11 | MNCN/ADN: 51930 | JX087535 | JX087463 | JX087597 |
|  |  |  | Washington | 01Dec10 | MNCN/ADN: 51931 | JX087536 | JX087464 | JX087598 |
|  | *Aeolidiella alba* Risbec, 1928 | *Bulbaeolidia* sp. A | Brazil | 16Oct09 | MZSP 97645 | - | JQ996803 | JQ996902 |
|  |  | *Bulbaeolidia* sp. A | Brazil | 16Oct09 | MZSP 97643 | JQ997011 | JQ996804 | JQ996903 |
|  |  | *Bulbaeolidia alba* (Risbec, 1928) | Japan | 10Oct11 | CASIZ 187743 | JQ997013 | JQ996806 | JQ996905 |
|  |  | *Bulbaeolidia alba* (Risbec, 1928) | Japan | 16Oct11 | CASIZ 187744 | JQ997014 | JQ996807 | JQ996906 |
|  |  | *Bulbaeolidia alba* (Risbec, 1928) | Japan | 21Oct11 | CASIZ 187745 | JQ997015 | JQ996808 | JQ996907 |
|  |  | *Bulbaeolidia alba* (Risbec, 1928) | Malaysia | 2Oct07 | CASIZ 176741 | JQ997016 | JQ996809 | JQ996908 |
|  |  | *Bulbaeolidia alba* (Risbec, 1928) | Philippines | 21May09 | CASIZ 180387 | JQ997012 | JQ996805 | JQ996904 |
|  |  | *Bulbaeolidia alba* (Risbec, 1928) | Philippines | 2011 | CASIZ 186207 | JQ997017 | JQ996810 | JQ996909 |
|  | *Aeolidiella alderi* (Cocks, 1852) |  | France (MED) | 26Jul02 | ZSM Mol 20020982 | HQ616765 | HQ616728 | HQ616794 |
|  |  |  | Italy | 18Apr01 | ZSM Mol 20012341 | HQ616766 | HQ616729 | HQ616795 |
|  |  |  | Italy | 18Apr01 | ZSM Mol 20012340 | - | JQ996811 | JQ996910 |
|  | *Aeolidiella chromosoma* Cockerell & Eliot, 1905 | *Anteaeolidiella chromosoma* (Cockerell &Eliot, 1905) | Mexico (EP) | 26Feb06 | CASIZ 173060 | JQ997018 | JQ996812 | JQ996911 |
|  |  |  | Peru | 04Dic06 | ZSM Mol 20090868 | - | JQ996813 | JQ996912 |
|  | *Aeolidiella* sp. A | *Anteaeolidiella* sp. B | Fiji | 20Aug06 | ZSM Mol 20070193 | JQ997020 | JQ996815 | JQ996914 |
|  | *Aeolidiella* sp. B | *Bulbaeolidia* sp. B | Hawaii | 15Oct09 | CASIZ 182172 | JQ997022 | JQ996817 | JQ996916 |
|  |  | *Bulbaeolidia* sp. B | Hawaii | 15Oct09 | CASIZ 182172 | JQ997023 | JQ996818 | JQ996917 |
|  | *Anteaeolidiella indica* (Bergh, 1888) | *Anteaeolidiella cacaotica* (Stimpson, 1855) | Eastern Australia | 14Feb10 | MNCN/ADN: 51922 | JX087528 | JX087455 | JX087590 |
|  |  | *Anteaeolidiella cacaotica* (Stimpson, 1855) | Japan | 10Aug10 | MNCN/ADN: 51924 | - | JX087457 | - |
|  |  | *Anteaeolidiella lurana* (Marcus &Marcus, 1967) | Bermuda | 20Jun09 | ZMBN82992 | - | JQ996821 | JQ996922 |
|  |  | *Anteaeolidiella lurana* (Marcus &Marcus, 1967) | Brazil | 19Dic07 | MZSP97638 | JQ997027 | JQ996822 | - |
|  |  | *Anteaeolidiella lurana* (Marcus &Marcus, 1967) | Brazil | 14Dec08 | MZSP97574 | - | - | JQ996923 |
|  |  | *Anteaeolidiella* sp. A | Clipperton Is. | 1998 | CASIZ 115686 | JQ997028 | JQ996823 | JQ996924 |
|  |  | *Anteaeolidiella* sp. A | Clipperton Is. | 1998 | CASIZ 115686 | JQ997029 | JQ996824 | JQ996925 |
|  |  | *Anteaeolidiella lurana* (Marcus &Marcus, 1967) | Italy | Oct11 | CASIZ 186820 | JQ997031 | JQ996826 | JQ996927 |
|  |  | *Anteaeolidiella takanosimensis* (Baba, 1930) | Japan | 19Mar06 | MNCN/ADN: 51923 | JX087529 | JX087456 | JX087591 |
|  |  | *Anteaeolidiella takanosimensis* (Baba, 1930) | Japan | 10Aug10 | MNCN/ADN: 51925 | JX087530 | JX087458 | JX087592 |
|  |  | *Anteaeolidiella cacaotica* (Stimpson, 1855) | Line Is. | 28Sep06 | CASIZ 174212 | JQ997030 | JQ996825 | JQ996926 |
|  |  | *Anteaeolidiella saldanhensis* (Barnard, 1927) | South Africa (EA) | 06Jan08 | CASIZ 176313 | JQ997032 | JQ996827 | JQ996928 |
|  | *Aeolidiella japonica* Eliot, 1913 | *Bulbaeolidia japonica* (Eliot, 1913) | Japan | 10Feb10 | CASIZ 184527 | JQ997033 | JQ996828 | JQ996929 |
|  | *Aeolidiella oliviae* MacFarland, 1966 | *Anteaeolidiella oliviae* (MacFarland, 1966) | California | 26May09 | CASIZ 181315 | JQ997034 | JQ996829 | JQ996930 |
|  | *Aeolidiopsis ransoni* Pruvot-Fol, 1956 | *Baeolidia ransoni* (Pruvot-Fol, 1956) | Philippines | 2011 | CASIZ 186208 | - | JQ996837 | JQ996938 |
|  |  |  | Philippines | 2011 | CASIZ 186209 | JQ997043 | JQ996838 | JQ996939 |
|  | *Aeolidiella sanguinea* (Norman, 1877) |  | France (EA) | 27Jun09 | MNCN/ADN: 51932 | JX087538 | JX087466 | JX087600 |
|  |  |  | France (EA) | 30Jun09 | MNCN/ADN: 51933 | JX087537 | JX087465 | JX087599 |
|  | *Aeolidiella stephanieae* Valdés, 2005 | *Berghia stephanieae* (Valdés, 2005) | Florida | 13Apr11 | CASIZ 185770 | JQ997044 | JQ996839 | JQ996940 |
|  | *Baeolidia* cf. *japonica* Baba, 1933 | *Baeolidia* sp. C | Marshall Is. | 11Oct10 | CASIZ 184503 | JQ997045 | JQ996840 | JQ996941 |
|  | *Baeolidia japonica* Baba, 1933 |  | Hawaii | 30Nov08 | CASIZ 180341 | - | JQ996854 | JQ996955 |
|  |  |  | Japan | 10Feb10 | CASIZ 184520 | JQ997058 | JQ996855 | JQ996956 |
|  |  |  | Marshall Is. | 12Sep11 | CASIZ 186795 | JQ997059 | JQ996856 | JQ996957 |
|  |  |  | Philippines | 27Dec08 | CASIZ 181357 | JQ997057 | JQ996853 | JQ996954 |
|  | *Baeolidia major* Eliot, 1903 | *Baeolidia moebii* Bergh, 1888 | Hawaii | 07May08 | MNCN 15.05/54987 | HQ616771 | HQ616734 | HQ616800 |
|  |  | *Baeolidia moebii* Bergh, 1888 | Hawaii | 4Oct08 | CASIZ 180327 | JQ997060 | JQ996857 | JQ996958 |
|  |  | *Baeolidia moebii* Bergh, 1888 | Marshall Is. | 11Feb08 | MNCN/ADN: 51948 | JX087550 | JX087481 | JX087618 |
|  |  | *Baeolidia moebii* Bergh, 1888 | Marshall Is. | 01Mar08 | MNCN/ADN: 51949 | JX087551 | JX087482 | JX087619 |
|  |  | *Baeolidia moebii* Bergh, 1888 | Philippines | 16Apr08 | CASIZ 177602 | HQ616770 | HQ616733 | HQ616799 |
|  |  | *Baeolidia moebii* Bergh, 1888 | Philippines | 04May11 | CASIZ 186211 | JQ997061 | JQ996858 | JQ996959 |
|  | *Baeolidia* sp. A |  | Marshall Is. | 24Jul11 | CASIZ 187741 | JQ997055 | JQ996850 | JQ996951 |
|  |  |  | Marshall Is. | 24Jul11 | CASIZ 187741 | JQ997056 | JQ996851 | JQ996952 |
|  |  |  | Philippines | 21May11 | CASIZ 186210 | JQ997054 | JQ996852 | JQ996953 |
|  |  |  | Philippines | 21Apr08 | CASIZ 177716 | JQ997051 | JQ996847 | JQ996948 |
|  | *Baeolidia* sp. B |  | Japan | 06Mar10 | CASIZ 184525 | JQ997046 | JQ996842 | JQ996943 |
|  | *Berghia coerulescens* (Laurillard, 1830) |  | Croatia | 03Dec04 | ZSM Mol 20041584 | JQ997049 | JQ996845 | JQ996946 |
|  |  |  | Spain (EA) | 26Mar09 | MNCN/ADN: 51952 | - | JX087470 | JX087604 |
|  | *Berghia columbina*  (García-Gómez & Thompson, 1990) |  | Morocco (EA) | 25Feb09 | MNCN/ADN: 51937 | - | JX087471 | JX087605 |
|  |  |  | Morocco (EA) | 28Mar09 | MNCN/ADN: 51938 | JX087542 | JX087472 | JX087606 |
|  |  |  | Morocco (EA) | 25Oct10 | MNCN/ADN: 51939 | JX087543 | JX087473 | JX087607 |
|  |  |  | Spain (EA) | 6Mar08 | MNCN/ADN: 51941 | JX087544 | - | JX087608 |
|  |  |  | Spain (EA) | 6Mar08 | MNCN/ADN: 51942 | JX087545 | JX087474 | JX087609 |
|  | *Berghia creutzbergi* Marcus & Marcus, 1970 |  | Bahamas | Oct09 | MNCN/ADN: 51943 | - | JX087475 | JX087612 |
|  |  |  | Bahamas | Oct09 | MNCN/ADN: 51944 | - | JX087476 | JX087613 |
|  |  |  | Colombia (WA) | 14Dec07 | MNCN/ADN: 51945 | JX087546 | JX087477 | JX087614 |
|  |  |  | Cuba | 16Jul08 | MNCN/ADN: 51946 | JX087547 | JX087478 | JX087615 |
|  | *Berghia* sp. A |  | Senegal | 8Jun05 | MNCN/ADN: 51947 | JX087549 | JX087480 | JX087617 |
|  | *Berghia rissodominguezi* Muniain & Ortea, 1999 |  | Colombia (WA) | Nov07 | MNCN/ADN: 51950 | - | JX087483 | JX087620 |
|  |  |  | Cuba | 26Jul08 | MNCN/ADN: 51951 | JX087552 | JX087484 | JX087621 |
|  | *Berghia verrucicornis* (A. Costa, 1864) |  | Morocco (EA) | 30Mar09 | MNCN 15.05/53686 | HQ616749 | HQ616712 | HQ616778 |
|  |  |  | Morocco (EA) | 25Oct10 | MNCN/ADN: 51953 | - | JX087485 | JX087622 |
|  |  |  | Senegal | 6Jun03 | MNCN/ADN: 51954 | - | JX087488 | JX087610 |
|  |  |  | Spain (EA) | 05Apr08 | MNCN 15.05/53687 | HQ616750 | HQ616713 | HQ616779 |
|  |  |  | Spain (EA) | 22Mar08 | MNCN/ADN: 51955 | JX087553 | JX087486 | JX087623 |
|  |  |  | Spain (EA) | 06Apr08 | MNCN/ADN: 51956 | JX087554 | JX087487 | JX087624 |
|  | *Cerberilla annulata* (Quoy & Gaimard, 1832) | *“Cerberilla” annulata* (Quoy &Gaimard, 1832) | Marshall Is. | 24Jul00 | CASIZ 182227 | - | JQ996866 | JQ996967 |
|  | *Cerberilla bernadettae* Tardy, 1965 | *“Cerberilla” bernadettae* Tardy, 1965 | Spain (EA) | 06Aprl08 | MNCN/ADN: 51957 | JX087555 | JX087489 | JX087625 |
|  | *Cerberilla* cf. *affinis* (Quoy & Gaimard, 1832) | *“Cerberilla”* cf. *affinis* (Quoy &Gaimard, 1832) | Philippines | 16May09 | CASIZ 180421 | - | JQ996863 | JQ996964 |
|  |  | *“Cerberilla”* cf. *affinis* (Quoy &Gaimard, 1832) | Philippines | 16May09 | CASIZ 180421 | JQ997065 | JQ996867 | JQ996968 |
|  | *Cerberilla* sp. 3 | *“Cerberilla”* sp. 3 | Hawaii | 28Nov07 | CASIZ 176794 | - | JQ996873 | JQ996976 |
|  | *Cerberilla* sp. A | *“Cerberilla* “sp. A | Philippines | 30Apr11 | CASIZ 186212 | - | - | JQ996975 |
|  |  | *“Cerberilla* “sp. A | Philippines | May10 | CASIZ 182900 | JQ997069 | - | JQ996977 |
|  |  | *“Cerberilla* “sp. A | Philippines | May10 | CASIZ 182900 | JQ997070 | JQ996874 | JQ996978 |
|  | *Cerberilla* sp. B | *“Cerberilla”* sp. B | Japan | 22Apr10 | CASIZ 187747 | JQ997068 | JQ996872 | JQ996973 |
|  | *Cerberilla* sp. C | *“Cerberilla”* sp. C | Japan | 12May10 | CASIZ 187748 | - | - | JQ996974 |
|  | *Limenandra fusiformis* (Baba, 1949) |  | Japan | 29Jul10 | CASIZ 184526 | JQ997077 | JQ996883 | JQ996988 |
|  |  |  | Japan | 29Jul10 | CASIZ 184526 | JQ997078 | JQ996884 | JQ996989 |
|  | *Limenandra nodosa* Haefelfinger & Stamm, 1958 |  | Bahamas | Sep09 | CASIZ 184521 | JQ997080 | JX087527 | JQ996991 |
|  |  |  | Bahamas | Sep09 | MNCN/ADN: 51958 | JX087559 | JX087493 | JX087629 |
|  |  |  | Balearic Is. (Spain, MED) | Sep2007 | MNCN/ADN 24.923 | HQ616768 | HQ616731 | HQ616797 |
|  |  |  | France (EA) | 21Jul10 | MNCN/ADN: 51959 | JX087560 | JX087494 | JX087630 |
|  |  |  | Madeira Is. (Portugal) | 18Sep11 | CASIZ 186792 | JQ997081 | JQ996886 | JQ996992 |
|  |  | *Limenandra* sp. A | Mexico (EP) | 27Feb06 | CASIZ 174108 | JQ997082 | JQ996887 | JQ996993 |
|  |  | *Limenandra* sp. A | Philippines | 21May09 | CASIZ 181280 | HQ616769 | HQ616732 | HQ616798 |
|  | *Limenandra* sp. B |  | Marshall Is. | 01Mar08 | MNCN/ADN: 51934 | JX087539 | JX087467 | JX087601 |
|  |  |  | Marshall Is. | 23Feb09 | CASIZ 181363 | JQ997050 | JQ996846 | JQ996947 |
|  |  |  | Marshall Is. | 25May09 | MNCN/ADN: 51935 | JX087540 | JX087468 | JX087602 |
|  |  |  | Marshall Is. | 30Aug09 | MNCN/ADN: 51936 | JX087541 | JX087469 | JX087603 |
|  | *Limenandra* sp. C |  | Philippines | 19Apr08 | CASIZ 177682 | - | JQ996841 | JQ996942 |
|  |  |  | Philippines | 16May10 | CASIZ 182761 | JQ997079 | JQ996885 | JQ996990 |
|  | *Spurilla neapolitana* (Delle Chiaje, 1841) |  | Azores Is. | 20Aug07 | MNCN/ADN: 51960 | JX087566 | JX087502 | JX087637 |
|  |  |  | Balearic Is. (Spain, MED) | 25May08 | MNCN/ADN: 51961 | JX087582 | JX087517 | JX087655 |
|  |  | *Spurilla braziliana* MacFarland, 1909 | Brazil | 16Aug07 | MNCN/ADN: 51984 | JX087567 | JX087503 | JX087638 |
|  |  | *Spurilla braziliana* MacFarland, 1909 | Brazil | 16Aug07 | MNCN/ADN: 51985 | JX087568 | - | JX087639 |
|  |  |  | Cape Verde | 01May11 | MNCN/ADN: 51962 | JX087569 | JX087504 | JX087640 |
|  |  |  | Cape Verde | 01May11 | MNCN/ADN: 51963 | JX087570 | JX087505 | JX087641 |
|  |  |  | Cape Verde | 01May11 | MNCN/ADN: 51964 | JX087571 | JX087506 | JX087642 |
|  |  |  | Cape Verde | 01May11 | MNCN/ADN: 51965 | JX087572 | JX087507 | JX087643 |
|  |  |  | Cape Verde | 2009 | MNCN/ADN: 51966 | JX087573 | - | - |
|  |  | *Spurilla braziliana* MacFarland, 1909 | Colombia (WA) | - | MNCN/ADN: 51986 | JX087575 | JX087508 | JX087644 |
|  |  | *Spurilla braziliana* MacFarland, 1909 | Costa Rica (EP) | 01Jan01 | CASIZ 175735 | JQ997095 | JQ996900 | - |
|  |  | *Spurilla braziliana* MacFarland, 1909 | Costa Rica (EP) | 13Jan01 | CASIZ 175737 | JQ997096 | - | JQ997009 |
|  |  |  | Croatia | 15Apr08 | MNCN/ADN: 51967 | JX087576 | JX087509 | JX087645 |
|  |  |  | Croatia | 09Dec07 | MNCN/ADN: 51968 | JX087577 | JX087510 | JX087646 |
|  |  | *Spurilla braziliana* MacFarland, 1909 | Cuba | 16Jul08 | MNCN/ADN: 51987 | JX087578 | JX087511 | JX087647 |
|  |  |  | France (EA) | 16Aug06 | MNCN/ADN: 51969 | JX087574 | JX087514 | JX087650 |
|  |  |  | France (EA) | 16Aug06 | MNCN/ADN: 51970 | - | JX087515 | JX087651 |
|  |  |  | France (EA) | 16Aug06 | MNCN/ADN: 51971 | - | - | JX087652 |
|  |  |  | France (EA) | 16Aug06 | MNCN/ADN: 51972 | - | - | JX087653 |
|  |  |  | France (EA) | 18Apr09 | MNCN/ADN: 51919 | - | JX114842 | JX114845 |
|  |  |  | Greece | 06Sep10 | MNCN/ADN: 51973 | JX087581 | JX087516 | JX087654 |
|  |  |  | Italy | 17Mar09 | MNCN/ADN: 51974 | JX087583 | - | JX087656 |
|  |  |  | Italy | 17Mar09 | MNCN/ADN: 51975 | - | JX087520 | JX087658 |
|  |  |  | Italy | 17Mar09 | MNCN/ADN: 51976 | JX087584 | JX087521 | JX087659 |
|  |  |  | Italy | 1Apr10 | MNCN/ADN: 51977 | - | JX087518 | - |
|  |  |  | Italy | 1Apr10 | MNCN/ADN: 51978 | - | JX087519 | JX087657 |
|  |  |  | Italy | 29Sep08 | MNCN 15.05/54988 | HQ616763 | HQ616726 | HQ616792 |
|  |  |  | Italy | 29Sep08 | MNCN/ADN: 51979 | JX087587 | JX087524 | JX087662 |
|  |  | *Spurilla braziliana* MacFarland, 1909 | Japan | 19Apr06 | MNCN/ADN: 51921 | - | - | JX114844 |
|  |  | *Spurilla braziliana* MacFarland, 1909 | Japan | 6Jul11 | CASIZ 187750 | JQ997097 | - | - |
|  |  |  | Morocco (EA) | 26Feb09 | MNCN/ADN: 51980 | - | JX087499 | - |
|  |  |  | Morocco (EA) | 28Mar09 | MNCN/ADN: 51981 | JX087564 | JX087500 | JX087635 |
|  |  |  | Morocco (EA) | 22Jul09 | MNCN/ADN: 51982 | JX087586 | JX087523 | JX087661 |
|  |  | *Spurilla* sp. A | Morocco (EA) | 23Jul09 | MNCN/ADN: 51988 | JX087565 | JX087501 | JX087636 |
|  |  |  | Portugal | 08Jul02 | CASIZ 175756 | HQ616764 | HQ616727 | HQ616793 |
|  |  |  | Portugal | 29Aug10 | MNCN/ADN: 51983 | JX087585 | JX087522 | JX087660 |
|  |  | *Spurilla* sp. A | Spain (EA) | Abr09 | MNCN/ADN: 51989 | JX087579 | JX087512 | JX087648 |
|  |  | *Spurilla* sp. A | Spain (EA) | Abr09 | MNCN/ADN: 51990 | JX087580 | JX087513 | JX087649 |
|  |  |  | Spain (EA) | 13Apr10 | MNCN/ADN: 51920 | - | JX114843 | - |
|  | *Spurilla* *salaamica* Rudman, 1982 | *Baeolidia* *salaamica* (Rudman, 1982) | Hawaii | 07Oct04 | CASIZ 180330 | JQ997048 | JQ996844 | JQ996945 |
|  |  | *Baeolidia* *salaamica* (Rudman, 1982) | Philippines | 17Mar04 | CASIZ 177397 | JQ997047 | JQ996843 | JQ996944 |
|  |  | *Baeolidia salaamica*  (Rudman, 1982) | Japan | 19Apr06 | CASIZ 184524 | - | JQ996862 | JQ996962 |
|  |  | *Baeolidia salaamica*  (Rudman, 1982) | Philippines | 16Apr08 | CASIZ 177599 | - | JQ996859 | - |
|  |  | *Baeolidia salaamica*  (Rudman, 1982) | Philippines | 16Apr08 | CASIZ 177599 | JQ997062 | JQ996860 | JQ996960 |
|  | *Spurilla* sp*.* A | *Spurilla sargassicola* (Kröyer in Bergh, 1861) | Bahamas | Sep09 | MNCN/ADN: 51991 | - | JX087525 | JX087663 |
|  |  | *Spurilla sargassicola* (Kröyer in Bergh, 1861) | Bahamas | Sep09 | CASIZ 184523 | JQ997098 | JQ996901 | JQ997010 |
|  |  | *Spurilla sargassicola* (Kröyer in Bergh, 1861) | Bahamas | Sep09 | MNCN/ADN: 51992 | JX087588 | - | JX087664 |
|  |  | *Spurilla sargassicola* (Kröyer in Bergh, 1861) | Bahamas | Sep09 | MNCN/ADN: 51993 | JX087589 | JX087526 | JX087665 |
| Babakinidae Roller, 1973 | *Babakina anadoni* (Ortea, 1979) |  | Bahamas | 29Jun99 | MNCN 15.05/46706 | - | - | HQ616805 |
|  |  |  | Brazil | May06 | MNRJ 10890 | - | HQ616742 | HQ616806 |
|  |  |  | Brazil | May06 | MNRJ 10891 | - | HQ616743 | - |
|  |  |  | Brazil | Feb06 | MNRJ 10893 | HQ616746 | HQ616709 | HQ616775 |
|  |  |  | Canary Is. (Spain, EA) | 30Oct04 | MNCN 15.05/46705 | HQ616747 | HQ616710 | HQ616776 |
|  |  |  | Spain (EA) | 25May05 | MNCN15.05/46704 | HQ616767 | HQ616730 | HQ616796 |
|  |  |  | Spain (EA) | 31Mar06 | MNCN 15.05/46979 | - | HQ616744 | HQ616807 |
|  |  |  | Spain (EA) | 5Sep03 | MNCN 15.05/46702 | HQ616748 | HQ616711 | HQ616777 |
|  | *Babakina festiva* (Roller, 1972) |  | California | 1Nov09 | CASIZ 182204 | - | HQ616735 | HQ616801 |
|  |  |  | California | 10Jan10 | CASIZ 182205 | - | HQ616736 | HQ616802 |
|  |  |  | Japan | May05 | MNCN 15.05/46741 | - | - | HQ616803 |
|  | *Babakina indopacifica* Gosliner,  González-Duarte & Cervera, 2007 |  | Philippines (GB) | 20Mar08 | - | HM162754 | HM162678 | HM162587 |
| Facelinidae Bergh, 1889 | *Caloria elegans* (Alder & Hancock, 1845) |  | Balearic Is. (Spain, MED) | 03May08 | MNCN 15.05/53689 | HQ616751 | HQ616714 | HQ616780 |
|  |  |  | Spain (EA) | 05Sep03 | MNCN 15.05/53690 | - | HQ616738 | - |
|  | *Caloria indica* (Bergh, 1896) |  | Hawaii (GB) | - | - | DQ417325 | DQ417273 | - |
|  | *Caloria militaris* (Alder & Hancock, 1864) |  | Philippines | 04May11 | CASIZ 186009 | - | JQ996875 | JQ996979 |
|  | *Caloria* sp. 4 |  | Philippines | 06May11 | CASIZ 186240 | JQ997063 | JQ996864 | JQ996965 |
|  |  |  | Philippines | 13May11 | CASIZ 186241 | JQ997064 | JQ996865 | JQ996966 |
|  | *Cratena peregrina* Gmelin, 1791 |  | Senegal | 30May05 | MNCN 15.05/53691 | HQ616752 | HQ616715 | HQ616781 |
|  |  |  | Spain (MED, GB) | - |  | AF249786 | - | - |
|  | *Dicata odhneri* Schmekel, 1967 |  | Spain (EA) | 02Aug06 | MNCN 15.05/53692 | HQ616773 | HQ616739 | - |
|  | *Dondice banyulensis* Portmann & Sandmeier, 1960 |  | Spain (EA) | 26May09 | MNCN 15.05/53693 | - | HQ616740 | HQ616804 |
|  |  |  | Spain (MED, GB) | - | - | AF249782 | - | - |
|  | *Facelina annulicornis* (Chamisso & Eysenhardt, 1821) |  | Azores Is. (Portugal) | 11Jun02 | CASIZ 186793 | JQ997076 | JQ996881 | JQ996986 |
|  |  |  | Balearic Is. (Spain, MED) | 26May08 | CASIZ 186794 | - | - | JQ996987 |
|  | *Facelina* sp. A |  | Philippines | 19May06 | CASIZ 182811 | JQ997052 | JQ996848 | JQ996949 |
|  | *Facelina* sp. B |  | Philippines | 19May09 | CASIZ 186213 | - | JQ996868 | JQ996969 |
|  |  |  | Philippines | May10 | CASIZ 182903 | - | JQ996882 | - |
|  |  |  | Philippines | 27Feb11 | CASIZ 186216 | - | JQ996871 | JQ996972 |
|  |  |  | Philippines | Aprl11 | CASIZ 186214 | JQ997066 | JQ996869 | JQ996970 |
|  |  |  | Philippines | Aprl11 | CASIZ 186214 | JQ997067 | JQ996870 | JQ996971 |
|  | *Facelina* sp. C |  | Philippines | 30Apr11 | CASIZ 186250 | JQ997092 | JQ996897 | JQ997004 |
|  |  |  | Philippines | 04May11 | CASIZ 186251 | JQ997093 | JQ996898 | JQ997005 |
|  |  |  | Philippines | 16May2010 | CASIZ 182750 | JQ997073 | JQ996877 | JQ996982 |
|  |  |  | Philippines | 17May2010 | CASIZ 182773 | JQ997072 | JQ996876 | JQ996981 |
|  | *Facelina* sp. D |  | Philippines | 01May11 | CASIZ 186244 | JQ997074 | JQ996878 | JQ996983 |
|  | Facelinid sp. 2 |  | Philippines | 01May11 | CASIZ 186258 | JQ997075 | JQ996879 | JQ996984 |
|  |  |  | Philippines | 10May11 | CASIZ 186008 | JQ997071 | JQ996880 | JQ996985 |
|  | Facelinid sp. A |  | Philippines | 10Mar11 | CASIZ 186437 | JQ997025 | - | JQ996920 |
|  | *Favorinus branchialis*  (Rathke, 1806) |  | Azores Is. (Portugal) | 11Jun02 | MNCN15.05/53694 | - | HQ616741 | - |
|  |  |  | Spain (EA) | 26Jun07 | MNCN 15.05/53695 | HQ616761 | HQ616724 | HQ616790 |
|  |  |  | Scotland (GB) | - | - | AY345042 | - | - |
|  | *Favorinus elenalexiarum* García & Troncoso, 2001 |  | Costa Rica (EP, GB) | 17Apr07 | - | HM162755 | HM162679 | HM162588 |
|  | *Godiva quadricolor*  (Barnard, 1927) |  | South Africa (EA, GB) | 09Jan08 | - | HM162692 | HM162602 | HM162508 |
|  | *Moridilla brockii* Bergh, 1888 |  | Philippines | 29Apri11 | CASIZ 186245 | JQ997083 | JQ996888 | JQ996994 |
|  | *Noumeaella isa* Marcus & Marcus, 1970 |  | Philippines | 01May11 | CASIZ 186249 | JQ997084 | JQ996889 | JQ996995 |
|  | *Noumeaella rehderi* Marcus, 1965 |  | Indonesia | 25Jul03 | ZSM Mol 20033794 | - | JQ996861 | JQ996961 |
|  | *Noumeaella* sp. 3 |  | Philippines | 13May11 | CASIZ 186006 | JQ997087 | JQ996892 | JQ996998 |
|  |  |  | Philippines | 13May11 | CASIZ 186006 | JQ997088 | JQ996893 | JQ996999 |
|  | *Noumeaella* sp. 4 |  | Philippines | 01May11 | CASIZ 186246 | JQ997090 | JQ996895 | JQ997002 |
|  |  |  | Philippines | 01May11 | CASIZ 186246 | JQ997091 | JQ996896 | JQ997003 |
|  |  |  | Philippines | 10May11 | CASIZ 186248 | JQ997089 | JQ996894 | JQ997000 |
|  |  |  | Philippines | 10May11 | CASIZ 186005 | - | - | JQ997001 |
|  |  |  | Philippines | 24May11 | CASIZ 186247 | JQ997085 | JQ996890 | JQ996996 |
|  |  |  | Philippines | 24May11 | CASIZ 186247 | JQ997086 | JQ996891 | JQ996997 |
|  | *Noumeaella* sp. A |  | Malaysia | 28Sep03 | CASIZ 176743 | JQ997053 | JQ996849 | JQ996950 |
|  | *Noumeaella* sp. B |  | Morocco (EA) | 8Nov10 | MNCN/ADN: 51996 | JX087548 | JX087479 | JX087616 |
|  | *Phidiana lynceus* Bergh, 1867 |  | Cuba | 17Jul08 | MNCN/ADN: 51994 | JX087563 | JX087498 | JX087634 |
|  |  |  | Cuba | 21Jul08 | MNCN/ADN: 51995 | JX087562 | JX087497 | JX087633 |
|  | *Phyllodesmium horridum*  (Macnae, 1954) |  | South Africa (EA, GB) | 03Jan08 | - | HM162757 | HM162681 | HM162590 |
|  | *Pleurolidia juliae* Burn, 1966 |  | Philippines | 05May05 | CASIZ 186217 | JQ997094 | JQ996899 | JQ997007 |
|  | *Pruvotfolia longicirrha* (Eliot, 1906) |  | Cape Verde | Mar10 | MNCN 15.05/53703 | HQ616760 | HQ616723 | HQ616789 |
|  | *Pruvotfolia pselliotes* (Labbé, 1923) |  | France (EA) | 05Sep04 | MNCN 15.05/53705 | HQ616762 | HQ616725 | HQ616791 |
|  | *Pruvotfolia* sp. A |  | Philippines | 01May11 | CASIZ 186257 | - | - | JQ997008 |
|  | *Pruvotfolia* sp. B |  | Philippines | 30April11 | CASIZ 186243 | - | - | JQ996980 |
|  | *Pteraeolidia ianthina* (Angas, 1864) |  | Philippines | 17Mar08 | CASIZ 177282 | - | - | JQ997006 |
|  | *Sakuraeolis enosimensis* (Baba, 1930) |  | California (GB) | 13Dec07 | - | HM162758 | HM162682 | HM162591 |
| Fionidae Alder &Hancock, 1855 | *Fiona pinnata* (Eschscholtz, 1831) |  | Morocco (EA) | 22Dec10 | MNCN/ADN: 51997 | JX087558 | JX087492 | JX087628 |
| Flabellinidae Bergh, 1881 | *Calmella cavolini* (Vérany, 1846) |  | Italy | Jul2004 | MNCN 15.05/53688 | HQ616772 | HQ616737 | - |
|  | *Flabellina affinis* (Gmelin, 1791) |  | Balearic Is. (Spain, MED) | 14Jul07 | MNCN 15.05/53696 | HQ616753 | HQ616716 | HQ616782 |
|  |  |  | Spain (MED, GB) | - | - | AY345055 | - | - |
|  |  |  | Spain (MED, GB) | - | - | AF249783 | - | - |
|  | *Flabellina babai* Schmekel, 1972 |  | Chafarinas Is. (MED) | 25Feb07 | MNCN 15.05/53698 | HQ616754 | HQ616717 | HQ616783 |
|  | *Flabellina baetica* García-Gómez, 1984 |  | Spain (EA) | 14Jan05 | MNCN 15.05/53699 | HQ616755 | HQ616718 | HQ616784 |
|  | *Flabellina confusa* Duarte, Cervera & Poddubetskaia, 2008 |  | France (ATL) | 27Aug09 | MNCN/ADN: 51998 | JX087557 | JX087491 | JX087627 |
|  |  |  | France (ATL) | 10Sep2009 | MNCN/ADN: 51999 | JX087556 | JX087490 | JX087626 |
|  | *Flabellina ischitana* Hirano & Thompson, 1990 |  | Spain (MED, GB) | - |  | AF249814 | - | - |
|  |  |  | Morocco (EA) | 07Mar08 | MNCN 15.05/53700 | HQ616756 | HQ616719 | HQ616785 |
|  |  |  | Spain (EA) | 14Jun08 | MNCN 15.05/53701 | - | H1616745 | HQ616808 |
|  |  |  | Spain (EA) | 26Mar09 | MNCN 15.05/53697 | HQ616757 | HQ616720 | HQ616786 |
|  | *Flabellina pedata* (Montagu, 1815) |  | Spain (MED) | 13Oct07 | MNCN 15.05/53702 | HQ616758 | HQ616721 | HQ616787 |
| Piseinotecidae Edmunds, 1970 | *Piseinotecus gabinieri* (Vicente, 1975) |  | Spain (MED) | 13Oct07 | MNCN/ADN: 52000 | JX087561 | JX087495 | JX087631 |
|  |  |  | Spain (MED) | 13Oct07 | MNCN/ADN: 52001 | - | JX087496 | JX087632 |
|  | *Piseinotecus gaditanus* Cervera, García-Gómez & García, 1987 |  | Spain (EA) | 20Jun07 | MNCN 15.05/53704 | HQ616759 | HQ616722 | HQ616788 |
|  | *Piseinotecus* sp. |  | Philippines (GB) | 22Apr08 | - | HM162694 | HM162604 | HM162510 |
| Tergipedidae Bergh, 1889 | *Catriona* sp. A |  | Hawaii | 05May08 | CASIZ 180288 | JQ997021 | JQ996816 | JQ996915 |
|  | *Catriona* sp. B |  | Peru | 09Dic06 | ZSM Mol 20090736 | JQ997024 | JQ996819 | JQ996918 |
|  |  |  | Peru | 09Dic06 | ZSM Mol 20090735 | - | - | JQ996919 |
|  | *Cuthona* sp. 35 |  | Philippines | 10Mar11 | CASIZ 186436 | JQ997026 | JQ996820 | JQ996921 |
|  | *Cuthona* sp. A |  | Philippines | 11Mar11 | CASIZ 186435 | JQ997019 | JQ996814 | JQ996913 |
