# Supplementary material for: A Tale That Morphology Fails to Tell: A Molecular Phylogeny of Aeolidiidae (Aeolidida, Nudibranchia, Gastropoda)
Source: PLoS One. 2013 May 2;8(5):e63000. doi: 10.1371/journal.pone.0063000 (PMC3642091; doi:10.1371/journal.pone.0063000)
Supplement: Table S2 — Minimum COI gene pairwise uncorrected p -distances between sister species of each genus. (DOCX) [file pone.0063000.s004.docx]

| **Genus** | **COI genetic distances (%)** |
| --- | --- |
| *Berghia* | 5.6 |
| *Spurilla* | 6.4 |
| *Limenandra* | 7.3 |
| *Aeolidia* | 8.3 |
| *Anteaeolidiella* | 8.4 |
| *Baeolidia* | 12 |
| *Bulbaeolidia* gen. nov. | 13.5 |
| *Aeolidiella* | 14.6 |
| *Cerberilla* | 16 |
