# Supplementary material for: A Tale That Morphology Fails to Tell: A Molecular Phylogeny of Aeolidiidae (Aeolidida, Nudibranchia, Gastropoda)
Source: PLoS One. 2013 May 2;8(5):e63000. doi: 10.1371/journal.pone.0063000 (PMC3642091; doi:10.1371/journal.pone.0063000)
Supplement: Table S3 — List of nominal species of Aeolidiidae. Final identifications are determined after a review of the pertinent literature. ✓ = species analysed here; X = species not found properly preserved for molecular analyses; − = validity and/or identity of this species not studied here. (DOCX) [file pone.0063000.s005.docx]

| **Genera** | **Nominal species** | **Genera attributed to** | **Studied in present work** | **Final ids** |
| --- | --- | --- | --- | --- |
| *Aeolidia* | *Aeolidia collaris* Odhner, 1921 | *Aeolidia* | X | - |
|  | *Aeolidia helicochorda* Miller, 1987 | *Aeolidia, Burnaia* | X | - |
|  | *Aeolidia herculea* Bergh, 1894 | *Aeolidia* | X | - |
|  | *Aeolidia farallonensis* Gosliner & Behrens 1996 | *Aeolidia* | X | Junior synonym of *Aeolidia herculea* |
|  | *Aeolidia papillosa* var. *pacifica* Bergh, 1879 | *Aeolidia* | X | Junior synonym of *Aeolidia papillosa* |
|  | *Aeolidia serotina* Bergh, 1873 | *Aeolidia* | X | Junior synonym of *Aeolidia papillosa* |
| *Aeolidiella* | *Aeolidiella alba* Risbec, 1928 | *Aeolidiella, Spurilla* | ✓ | *Bulbaeolidia alba* |
|  | *Aeolidiella albopunctata* Lin, 1992 | *Aeolidiella* | ✓ | - |
|  | *Aeolidiella bassethulli* Risbec, 1928 | *Aeolidiella* | ✓ | Junior synonym of *Anteaeolidiella cacaotica* |
|  | *Aeolidiella berghia* Baba, 1937 | *Aeolidiella* | X | - |
|  | *Aeolidiella croisicensis* Labbé, 1923 | *Aeolidiella* | X | - |
|  | *Aeolidiella drusilla* Bergh, 1900 | *Aeolidiella* | X | - |
|  | *Aeolidiella faustina* Bergh, 1900 | *Aeolidiella, Spurilla* | X | *-* |
|  | *Aeolidiella hulli* Risbec, 1928 | *Aeolidiella* | ✓ | Junior synonym of *Anteaeolidiella cacaotica* |
|  | *Aeolidiella indica* Bergh, 1888 | *Aeolidiella, Antaeolidiella* | X | *-* |
|  | *Aeolidiella japonica* Eliot, 1913 | *Aeolidiella, Spurilla* | ✓ | *Bulbaeolidia japonica* |
|  | *Aeolidiella lurana* Marcus & Marcus, 1967 | *Aeolidiella* | ✓ | *Anteaeolidiella lurana* |
|  | *Aeolidiella multicolor* Macnae, 1954 | *Aeolidiella* | ✓ | Junior synonym of *Anteaeolidiella saldanhesis* |
|  | *Aeolidiella occidentalis* Bergh, 1874 | *Aeolidiella* | X | - |
|  | *Aeolidiella oliviae* MacFarland, 1966 | *Aeolidiella, Spurilla* | ✓ | *Anteaeolidiella oliviae* |
|  | *Aeolidiella orientalis* Bergh, 1888 | *Aeolidiella, Spurilla* | X | - |
|  | *Aeolidiella orientalis takanosimensis* Baba, 1930 | *Aeolidiella* | ✓ | Junior synonym of *Anteaeolidiella takanosimensis* |
|  | *Aeolidiella saldanhesis* Barnard, 1927 | *Aeolidiella* | ✓ | *Anteaeolidiella saldanhesis* |
|  | *Aeolidiella soemmeringii* var. *mediterranea* Bergh, 1885 | *Aeolidiella* | ✓ | Junior synonym of *Aeolidiella alderi* |
|  | *Aeolidiella stephanieae* Valdés, 2005 | *Aeolidiella* | ✓ | *Berghia stephanieae* |
| *Aeolidiopsis* | *Aeolidiopsis harrietae* Rudman, 1982 | *Aeolidiopsis, Baeolidia* | X | Likely *Baeolidia harrietae* |
|  | *Aeolidiopsis ransoni* Pruvot-Fol, 1956 | *Aeolidiopsis* | ✓ | *Baeolidia ransoni* |
| *Aeolis* | *Aeolis cacaotica* Stimpson, 1855 | *Aeolis* | ✓ | *Anteaeolidiella cacaotica* |
|  | *Aeolis foulisi* Angas, 1864 | *Aeolidiella, Aeolis, Antaeolidiella* | ✓ | Junior synonym of *Anteaeolidiella cacaotica* |
|  | *Aeolis grandis* Volodchenko, 1941 | *Aeolis* | X | Junior synonym of *Aeolidia herculea* |
|  | *Aeolis longibranchus* Volodchenko, 1941 | *Aeolis, Cerberilla* | X | *-* |
|  | *Aeolis macleayi* Angas, 1864 | *Aeolidiella, Spurilla* | X | - |
| *Baeolidia* | *Baeolidia benteva* Marcus, 1958 | *Aeolidiella, Baeolidia, Berghia* | X | - |
|  | *Baeolidia cryoporos* Bouchet, 1977 | *Aeolidia, Baeolidia* | X | *-* |
|  | *Baeolidia fusiformis*, Baba, 1949 | *Baeolidia, Limenandra* | ✓ | *Limenandra fusiformis* |
|  | *Baeolidia japonica* Baba, 1933 | *Baeolidia, Berghia* | ✓ | *Baeolidia japonica* |
|  | *Baeolidia major* Eliot, 1903 | *Baeolidia, Berghia, Spurilla* | ✓ | Junior synonym of *Baeolidia moebii* |
|  | *Baeolidia major amakusana* Baba, 1937 | *Baeolidia* | ✓ | Junior synonym of *Baeolidia moebii* |
|  | *Baeolidia moebii* Bergh, 1888 | *Baeolidia* | ✓ | *Baeolidia moebii* |
|  | *Baeolidia palythoae* Gosliner, 1985 | *Baeolidia* | X | Likely *Baeolidia palythoae* |
|  | *Baeolidia quoyi* Pruvot-Fol, 1934 | *Baeolidia, Spurilla* | X | - |
| *Berghia* | *Berghia chaka* Gosliner, 1985 | *Berghia* | X | Likely *Baeolidia chaka* |
|  | *Berghia creutzbergi* Marcus & Marcus, 1970 | *Berghia, Spurilla* | ✓ | *Berghia creutzbergi* |
|  | *Berghia dela* Marcus & Marcus, 1960 | *Berghia* | X | - |
|  | *Berghia marcusi* Domínguez, Troncoso & García 2008 | *Berghia* | X | - |
|  | *Berghia modesta* Trinchese, 1882 | *Berghia* | ✓ | Junior synonym of *Berghia coerulescens* |
|  | *Berghia norvegica* Odhner, 1939 | *Berghia* | X | - |
|  | *Berghia rissodominguezi* Muniain & Ortea, 1999 | *Berghia, Spurilla* | ✓ | *Berghia rissodominguezi* |
| *Cerberilla* | *Cerberilla affinis* Bergh, 1888 | *Cerberilla* | ✓ | *“Cerberilla” affinis* |
|  | *Cerberilla africana* Eliot 1903 | *Cerberilla* | X | - |
|  | *Cerberilla albopunctata* Baba, 1976 | *Cerberilla* | X | - |
|  | *Cerberilla ambonensis* Bergh, 1905 | *Cerberilla* | X | - |
|  | *Cerberilla asamusiensis* Baba, 1940 | *Cerberilla* | X | - |
|  | *Cerberilla bernadettae* Tardy, 1965 | *Cerberilla* | ✓ | *“Cerberilla” bernadettae* |
|  | *Cerberilla chavezi* Hermosillo & Valdés, 2007 | *Cerberilla* | X | - |
|  | *Cerberilla incola* Burn, 1974 | *Cerberilla* | X | - |
|  | *Cerberilla longicirrha* Bergh, 1873 | *Cerberilla* | X | - |
|  | *Cerberilla mosslandica* McDonald & Nybakken 1975 | *Cerberilla* | X | - |
|  | *Cerberilla portiguara* Padula & Delgado, 2010 | *Cerberilla* | X | - |
|  | *Cerberilla pungoarena* Collier & Farmer, 1964 | *Cerberilla* | X | - |
|  | *Cerberilla tanna* Marcus & Marcus, 1960 | *Cerberilla* | X | - |
| *Eolidia* | *Eolidia annulata* Quoy & Gaimard, 1832 | *Cerberilla, Eolidia* | ✓ | *“Cerberilla” annulata* |
|  | *Eolidia coerulescens* Laurillad, 1830 | *Berghia, Eolidia* | ✓ | *Berghia coerulescens* |
|  | *Eolidia soemmeringii* Leuckart, 1828 | *Aeolidiella, Eolidia* | ✓ | Junior synonym of *Aeolidiella alderi* |
| *Eolis* | *Eolis alderi* Cocks, 1852 | *Aeolidiella, Eolis* | ✓ | *Aeolidiella alderi* |
|  | *Eolis alderiana* Deshayes & Fredol, 1865 | *Eolis* | ✓ | Junior synonym of *Spurilla neapolitana* |
|  | *Eolis campbellii* Cunningham, 1871 | *Eolis* | X | Likely senior name of *Aeolidia serotina* |
|  | *Eolis glauca* Alder & Hancock, 1845 | *Aeolidiella, Eolis* | X | - |
|  | *Eolis grossularia* Fischer, 1869 | *Eolis* | ✓ | Junior synonym of *Berghia verrucicornis* |
|  | *Eolis neapolitana* delle Chiaje, 1841 | *Eolis, Spurilla* | ✓ | *Spurilla neapolitana* |
|  | *Eolis pallidula* Lafont, 1871 | *Eolis* | ✓ | Junior synonym of *Aeolidiella glauca* |
|  | *Eolis peregrina* delle Chiaje, 1841 | *Eolis* | ✓ | Junior synonym of *Berghia coerulescens* |
|  | *Eolis sanguinea* Norman, 1877 | *Aeolidiella, Eolis* | ✓ | *Aeolidiella sanguinea* |
| *Facelina* | *Facelina sargassicola* Kröyer in Bergh, 1861 | *Facelina, Spurilla* | ✓ | *Spurilla sargassicola* |
| *Fenrisia* | *Fenrisia moebii* Bergh, 1888 | *Cerberilla, Fenrisia* | X | - |
| *Flabellina* | *Flabellina inornata* A. Costa, 1866 | *Flabellina* | ✓ | Junior synonym of *Spurilla neapolitana* |
|  | *Flabellina verrucicornis* A. Costa, 1864 | *Berghia, Flabellina, Spurilla* | ✓ | *Berghia verrucicornis* |
| *Limax* | *Limax papillosus* Linnaeus, 1761 | *Aeolidia, Limax* | ✓ | *Aeolidia papillosa* |
| *Limenandra* | *Limenandra nodosa* Haefelfinger & Stamm, 1958 | *Baeolidia, Limenandra* | ✓ | *Limenandra nodosa* |
| *Milleraeolidia* | *Milleraeolidia ritmica* Ortea, Caballer & Espinosa, 2004 | *Milleraeolidia* | X | Likely a junior synonym of *Berghia creutzbergi* |
| *Pleurolidia* | *Pleurolidia juliae* Burn, 1966 | *Pleurolidia, Protaeolidiella* | ✓ | Not included in Aeolidiidae |
| *Protaeolidiella* | *Protaeolidiella atra* Baba, 1955 | *Protaeolidiella* | X | - |
| *Spurilla* | *Spurilla australis* Rudman, 1982 | *Berghia, Spurilla* | X | Likely *Baeolidia australis* |
|  | *Spurilla braziliana* MacFarland, 1909 | *Spurilla* | ✓ | *Spurilla braziliana* |
|  | *Spurilla chromosoma* Cockerell & Eliot, 1905 | *Aeolidiella, Spurilla* | ✓ | *Anteaeolidiella chromosoma* |
|  | *Spurilla columbina* García-Gómez & Thompson, 1990 | *Berghia, Spurilla* | ✓ | *Berghia columbina* |
|  | *Spurilla dakarensis* Pruvot-Fol, 1953 | *Spurilla* | X | *Nomen dubium* |
|  | *Spurilla gabriellae* Vannucci, 1952 | *Spurilla* | ✓ | Junior synonym of *Spurilla braziliana* |
|  | *Spurilla orientalis* Bergh, 1905 | *Spurilla* | X | - |
|  | *Spurilla margaritae* Labbé, 1923 | *Spurilla* | ✓ | Junior synonym of *Berghia verrucicornis* |
|  | *Spurilla mograbina* Pruvot-Fol, 1953 | *Spurilla* | X | - |
|  | *Spurilla risbeci* Marcus, 1961 | *Spurilla* | ✓ | Junior synonym of *Anteaeolidiella takanosimensis* |
|  | *Spurilla salaamica* Rudman, 1982 | *Berghia, Spurilla* | ✓ | *Baeolidia salaamica* |
|  | *Spurilla vayssierei* García-Gómez & Cervera, 1985 | *Spurilla* | ✓ | Junior synonym of *Spurilla neapolitana* |
